# Interplay of oxidative stress and antioxidant mechanisms in cancer development and progression

**DOI:** 10.1007/s00204-025-04146-5

**Published:** 2025-09-04

**Authors:** Klaudia Jomova, Suliman Y. Alomar, Richard Valko, Lukas Fresser, Eugenie Nepovimova, Kamil Kuca, Marian Valko

**Affiliations:** 1 Department of Chemistry, Faculty of Natural Sciences, Constantine the Philosopher University in Nitra, 949 74 Nitra, Slovakia; 2 Doping Research Chair, Zoology Department, College of Science, King Saud University, 11451 Riyadh, Saudi Arabia; 3 Department of Chemistry, Faculty of Sciences, University of Hradec Kralove, 50003 Hradec Kralove, Czech Republic; 4 Center of Advanced Innovation Technologies, VSB-Technical University of Ostrava, 708 00 Ostrava-Poruba, Czech Republic; 5 Biomedical Research Center, University Hospital Hradec Kralove, 5005 Hradec Kralove, Czech Republic; 6 Centre for Basic and Applied Research, Faculty of Informatics and Management, University of Hradec Kralove, 50003 Hradec Kralove, Czech Republic; 7 Faculty of Chemical and Food Technology, Slovak University of Technology, 812 37 Bratislava, Slovakia

**Keywords:** Cancer, ROS, Oxidative stress, Antioxidants, Cell signaling, Tumorigenesis

## Abstract

Cellular systems responsible for the formation and removal of reactive oxygen species (ROS), functioning within physiological limits, are essential for maintaining intracellular redox balance. This state is known as oxidative eustress. Key redox signaling molecules, such as superoxide anion radical (O_2_^•—^) and hydrogen peroxide (H_2_O_2_), operate at nanomolar concentrations and are produced by NADPH oxidases (regulated by various factors), the mitochondrial electron transport chain (ETC), and numerous enzymes. In addition, cell signaling is influenced by nitric oxide (NO^•^) and reactive lipid species. Disruption of ROS signaling can lead to oxidative stress, a harmful condition associated with many chronic diseases, including cancer. The dual nature of ROS is evident in premalignant and malignant cells at all stages of tumor development, including proliferation, migration/invasion, angiogenesis, inflammation, immune evasion, and metastasis. ROS can promote tumor formation by regulating immune cells, mitochondrial metabolism, DNA methylation, DNA damage [such as the DNA oxidation product, 8-oxo-dG, resulting from hydroxyl radical (^•^OH) attack], and other mechanisms. The tumor-promoting activity mediated by H_2_O_2_ manifests through the promotion of epithelial-to-mesenchymal transition (EMT) and the formation of the tumor microenvironment (TME) by tumor-associated macrophages. While ROS are vital for tumor initiation and growth, their excessive production can also have anticancer effects by inducing senescence, apoptosis, or necrosis. ROS-related anticancer mechanisms include mitochondrial dysfunction, p53-dependent apoptosis, iron-dependent ferroptosis, activation of endoplasmic reticulum stress, inhibition of growth signaling pathways (such as the epidermal growth factor pathway, EGF), among others. Tumor cells employ a range of adaptive mechanisms to effectively maintain ROS levels within a dynamic range that promotes proliferation while preventing cell death. This regulation is achieved by fine-tuning the effects of antioxidants throughout all stages of cancer. During early tumor development, characterized by increased oncogene-induced oxidative stress, cancer cells depend on glutathione (GSH) and upregulated antioxidant gene expression controlled by nuclear factor erythroid 2-related factor 2 (NRF2) to maintain redox balance. The opposing roles of certain antioxidant enzymes, such as Mn-SOD (SOD2), illustrate the same duality as ROS, acting as potential tumor suppressors during early carcinogenesis and as tumor promoters during metastasis. Low-molecular-weight antioxidants such as vitamins C (ascorbate) and E (tocopherols), carotenoids (e.g., lycopene, β-carotene), flavonoids (e.g., quercetin), and isoflavones demonstrate effective antioxidant activity in vitro, but their anticancer effects in clinical settings remain unproven. Understanding the influence of the antioxidant network and the redox threshold on epithelial-to-mesenchymal transition and key tumor microenvironment components could lead to more effective therapeutic strategies. This review explores the dual roles of ROS and antioxidants throughout different stages of cancer progression.

## Introduction

Cancer is the second leading cause of death worldwide, accounting for ~ 17% of all deaths in 2021 (Siegel et al. 2024). Cancer is a complex, multifactorial disease whose risk of development and progression depends on both intrinsic (oncogenic signaling, epigenetic alterations, genomic instability, and disturbed cell metabolism) and extrinsic (tissue damage, infections, and mutagenic species) factors. The heterogeneity of various cancers is well-documented by comparing adult and child cancers. While childhood malignancies are usually restricted to the brain and hematological malignancies with relatively low mutational loads, adults at an advanced age (over 60) develop cancer predominantly within epithelial tissue with a significant mutational burden (Jassim et al. 2023).

Individual theories of the origin of cancer assume the existence of specific elements necessary for transforming a normal cell into a cancerous one (Hanahan and Weinberg  2011). The importance and specifics of these elements depend on the cell and cancer type. The various malignancies that develop in adults and children reflect cell susceptibility and the nonrandom distribution of tumors throughout the body. Cell susceptibility to cancer is related to differences in exposure to risk factors and cell plasticity related to the stage of development, aging, or other factors, altering the epigenome of cells to a state that can be transformed.

Alterations in the DNA sequence can activate the function of oncogenes or inactivate tumor suppressor genes. A complex genetic analysis of a large number of samples revealed that approximately 1000 genes are linked to human cancers, comprising approximately 250 oncogenes and 700 tumor suppressors (Wishart 2015). Cells usually require more than two or more mutations in these cancer-associated genes to become carcinogenic, which subsequently generates many different cancer genotypes. Whole-genome sequencing of tumor samples requires the analysis of several tens of thousands of distinct single-nucleotide variations, which makes tumor cells a genetic"train wreck"(Lee et al. 2010).

Many oncogenes and tumor suppressor genes have been shown to be important for cellular metabolism pathways (Boroughs And DeBerardinis 2015), involving aerobic glycolysis (metabolic conversion of glucose into lactate even in the presence of oxygen), glutaminolysis (degradation of glutamine to gain cellular energy), and one-carbon metabolism (interlinking metabolic pathways that include the methionine and folate cycles) (Clare et al. 2019; Martinez-Reyes And Chandel 2021). Cancer cells utilize these pathways to produce nucleotides, amino acids, fatty acids, and other intermediates required for cell growth and proliferation.

Interestingly, before 1970, many scientists considered cancer to be a metabolic disease. The"Warburg effect"was published in 1927 and revealed that cancer cells have different metabolic phenotypes and can consume up to 200 times more glucose than normal cells can consume (Warburg et al. 1927). The identification of oncogenes in 1971 gradually shifted cancer research from a metabolic approach to a genetic approach. However, the last decade has seen a renaissance of the metabolic approach, mainly because of the development of metabolomics and the identification of oncometabolites important for the initiation of tumor growth and metastasis. 2-Hydroxyglutarate was one of the first identified oncometabolites found at high concentrations in gliomas (Ward et al. 2010).

Cancer cells are characterized by increased levels of free radicals or reactive oxygen species (ROS), which are associated with both genetic alterations and metabolic pathways. The concept of ROS-induced oxidative stress is characterized as"*an imbalance between oxidants and antioxidants that favors oxidants, resulting in a disturbance of redox signaling and control, as well as potential molecular damage*"(Sies 2019). Physiological oxidative stress, which occurs at low levels, plays a role in redox signaling and regulation and is referred to as oxidative eustress. Conversely, when oxidative challenges exceed physiological levels, they can disrupt redox signaling and cause oxidative damage to biomolecules, termed oxidative distress. A significant portion of contemporary research focuses on the implications of these phenomena in the origin of many chronic diseases, including cancer and aging.

DNA damage is widely acknowledged as a significant contributor to the onset and progression of cancer. DNA can be damaged by various endogenous and exogenous insults, including ROS, causing distinct forms of damage (Glorieux et al. 2024). These include DNA lesions, the most dangerous of which are double-strand breaks that can be mutagenic due to chromosomal rearrangements or genetic information loss due to incorrect DNA repair. The flux of metabolic processes is known to produce ROS but can also be a target of oxidant species. Hypoxia-induced energy supply and metabolic requirements could lead to an increased ROS response through the Ca^2+^ influx pathway.

With the advancement of current experimental methods and a thorough understanding of the genetic and epigenetic pathways underlying cell transformation on one hand and owing to progress in the field of cancer metabolism on the other hand, the interconnection of these two fields may, with some caution, provide effective anticancer strategies.

This review aims to examine the various aspects of oxidants in cancer biology and emphasize some of the intricate roles of ROS in tumor and mesenchymal stromal cells at various phases of tumor initiation, promotion, malignant transformation, and progression.

## Cellular sources of ROS production

Cells produce various ROS or reactive nitrogen species (RNS) through multiple mechanisms (Sies And Jones 2020). The most important sources of ROS include mitochondria and NADPH oxidases (NOX); the crosstalk between them may play an important role in redox signaling. Other sources of ROS include cytochrome P450, xanthine oxidase (XO), peroxidases, the endoplasmic reticulum, cyclooxygenases, and uncoupled nitric oxide (NO) synthase (de Almeida et al. 2022). The important ROS/RNS produced in biological systems include superoxide radical anions (O_2_^•—^), hydrogen peroxide (H_2_O_2_), hydroxyl radicals (^•^OH), nitric oxide (NO^•^), peroxynitrite (ONOO^—^), peroxyl/alkoxyl radicals (ROO^•^/RO^•^), hypochlorous acid (HClO), and many others.

### Mitochondrial dysfunction

A series of five main protein complexes integrated into the inner mitochondrial membrane form the mitochondrial electron transport chain (ETC), which utilizes electron-transfer reactions responsible for ATP generation via oxidative phosphorylation (Nolfi-Donegan et al. 2020; Kowaltowski et al. 2009). ETC’s main route of ROS formation is considered premature and accidental leakage of electrons from complexes I, II, and III to reduce molecular oxygen to superoxide radical anions (O_2_ + e^—^ → O_2_^•—^). The flux of O_2_^•—^ depends on the abundance of electron donors, the reaction rate at which this redox-active donor reacts with O_2_, the partial pressure of O_2_, the rate of ATP production, the flux of electrons, the proton motive force (Δp), and other conditions. According to earlier research, between 1% and 2% of electrons entering the ETC produce superoxide radicals (Boveris And Chance 1973).

Flavin mononucleotide (FMN), a cofactor of respiratory complex I, plays a key role in the electron transport chain (Murphy 2009). The production of O_2_^•—^ at the FMN center is driven by the NADH/NAD^+^ ratio and is relatively low under physiological conditions, when respiration and ATP production are high and the proton motive force (Δp) is low. However, when the demand for ATP is lower than that for NAD^+^, this condition may result in damage to the ETC, which in turn may trigger increased O_2_^•—^ production.

Complex III, known as coenzyme Q − cytochrome c oxidoreductase, also generates O_2_^•—^within the ETC. Complex III is a membrane protein complex that accepts electrons from ubiquinol (QH2) and transfers them to the electron carrier cytochrome c. The rate of ROS production at this site under physiological conditions is rather low. Unlike complexes I and III, complex II does not produce a large amount of O_2_^•—^. One would assume that a lower oxygen concentration leads to suppressed superoxide formation. Paradoxically, hypoxia increases mitochondrial ROS production (Waypa And Schumacker 2008).

Redox equilibrium in mitochondria is maintained primarily by manganese superoxide dismutase (Mn-SOD, SOD2), which is localized in the mitochondrial matrix and rapidly dismutates O_2_^•—^ to H_2_O_2_. Alternatively, O_2_^•—^ is dismutated by copper–zinc-SOD (Cu, Zn-SOD, SOD1) localized in the intermembrane space according to the reaction (Zhao et al. 2019):

1 $$2O_{2}^{ \bullet - } + {2H^{ + }}^{SOD}\! \!\to O_{2} + H_{2} O_{2}$$

Unlike O_2_^•—^, H_2_O_2_ is a neutral, stable, weak oxidant that can diffuse out of the mitochondria to the cytosol, where it can regulate a number of signaling pathways, such as apoptosis, phosphorylation signaling, cell growth, differentiation, and other important pathways (Shadel And Horvath 2015). The generation of H_2_O_2_ is triggered by growth factors, chemokines, or physical stressors, and its intracellular concentration is tightly controlled and maintained at the nanomolar level (up to a maximum of 100 nM) (Sies 2017). In addition, H_2_O_2_ can increase the level of oxidative stress, for example, via the Fe-catalyzed Fenton reaction, resulting in the formation of an oxidizing hydroxyl radical (^•^OH):

2 $$\normalsize Fe^{2 + } + H_{2} O_{2} \to Fe^{3 + } + ^{\bullet}OH + OH^{ - }$$

^•^OH is one of the most reactive and highly oxidizing radicals, with a half-life of approximately 1 ns in a biological environment. ^•^OH reacts with all important biomolecules, including nucleic acids (resulting in mutations), membrane lipids (resulting in lipid peroxidation), or protein amino acids (resulting in the modification of protein side chains), with high (~ 10^9^–10^10^ M^−1^ s^−1^) rate constants.

Increased ROS production in mitochondria under stress conditions is related to the activity of inflammatory cytokines. Tumor necrosis factor-α (TNF-α) negatively affects the activity of mitochondrial complex I, which may result in mitochondrial dysfunction and cell death (Prajapati et al. 2015).

### Nicotinamide adenine dinucleotide phosphate (NADPH) oxidases (NOX)

Another important source of ROS is the transmembrane protein nicotinamide adenine dinucleotide phosphate (NADPH) oxidase (NOX) family, which is regulated by cytokines and growth factors. The human genome encodes seven isoforms of NOX (NOX1–5, DUOX1, 2), which facilitate electron transfer from NADPH on one side across the plasma membrane to O_2_ on the other side, thus forming O_2_^•—^ or H_2_O_2_ (Chen et al. 2019 ). These seven isoforms of NOX differ in their location, activation, and type of ROS they produce (Fukai And Ushio-Fukai 2020). Whereas NOX1–3 and NOX5 produce O_2_^•—^, NOX4 is known to release H_2_O_2_. ROS formation by NADPH oxidase is triggered by toll-like receptor agonists, which stimulate the innate immune system, resulting in a proinflammatory response manifested by the release of proinflammatory cytokines, such as interleukin-6 (IL-6), tumor necrosis factor-alpha (TNF-α), tumor growth factor-beta (TGF-β), and platelet-derived growth factor (PDGF) (Jomova et al. 2025). In addition, the hormones angiotensin and aldosterone and the 21-amino acid peptide endothelin, which are involved in the regulation of blood pressure, can stimulate ROS production through NADPH oxidase (Krylatov et al. 2018).

It has been reported that overexpressed NOX1 and NOX4 are involved in the senescence of endothelial cells (Lee et al. 2020; Sfeir et al. 2009). This finding was supported by NOX4 knockout rats, which do not exhibit these characteristics (Lee et al. 2020). Thus, ROS regulation by NOX may represent one of the key mechanisms involved in the incidence of age-related chronic diseases. NOX4 has also been confirmed to be a key regulator of the increased expression of proinflammatory mediators in vascular smooth muscle cells (Lozhkin et al. 2017). In addition to ROS production, NADPH is necessary for many other cellular processes, such as fatty acid, nucleotide, and proline synthesis (Chen et al. 2019).

### Crosstalk between mitochondria and NADPH oxidase

**Fig. 1:**
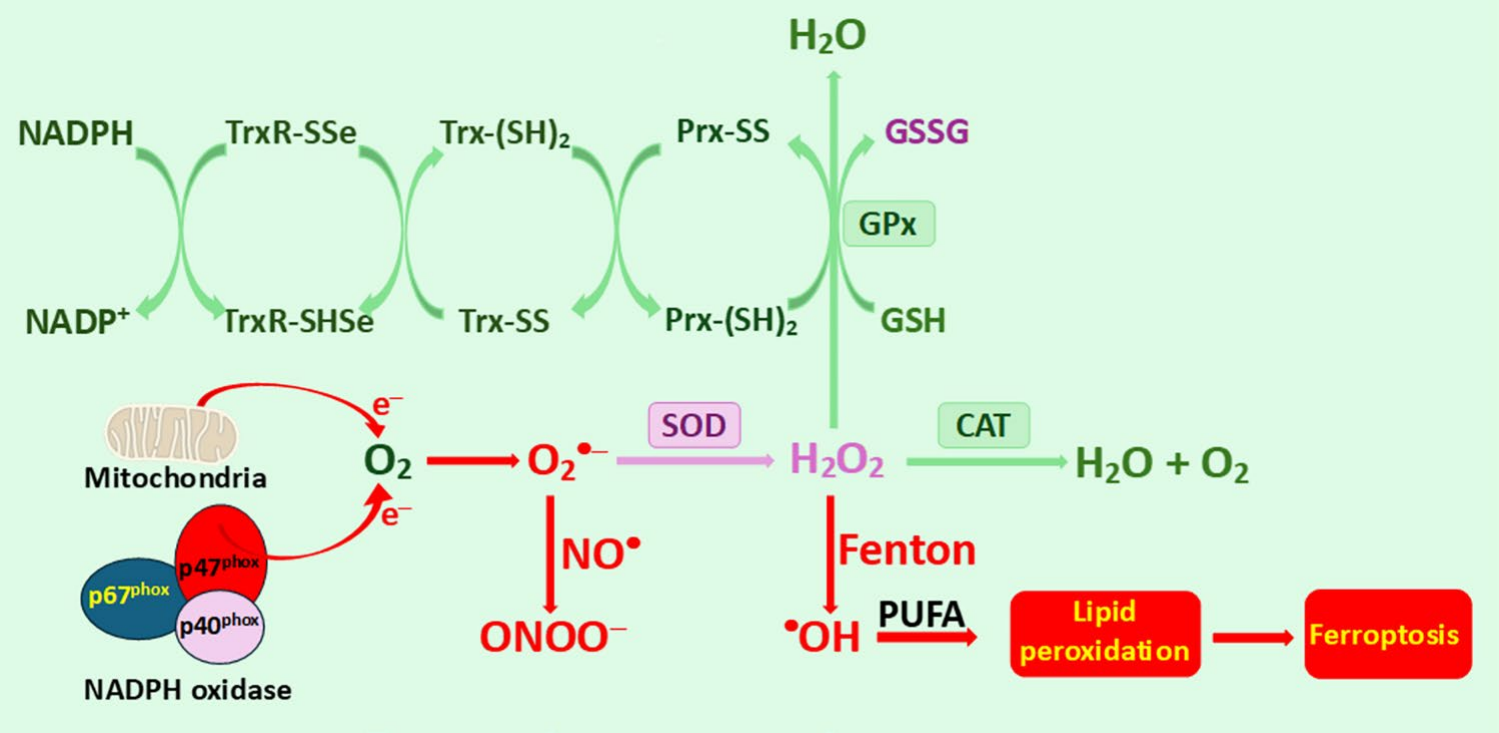
Schematic view of the generation and transformation of ROS. ROS generation primarily occurs through the release of electrons from the mitochondrial electron transport chain and NADPH oxidase (NOX) activity. Leaked electrons may interact with molecular oxygen, producing superoxide radical anion (O_2_^•—^), the precursor to other ROS. Superoxide can react very quickly with the transient molecule nitric oxide (NO^•^) to form peroxynitrite (ONOO^—^), a highly reactive compound that can alter the structure and function of proteins. Alternatively, the antioxidant enzyme superoxide dismutase (SOD) can catalyze the conversion of O_2_^•—^ into hydrogen peroxide (H_2_O_2_), which may undergo further transformations. In the presence of traces of redox metals, such as Fe^2+^, the Fenton reaction can generate highly reactive hydroxyl radicals (^•^OH) that damage lipids, proteins, and DNA. During ferroptosis, polyunsaturated fatty acids (PUFAs) are the main substrate of lipid peroxidation by hydroxyl radicals (•OH). The antioxidant enzyme catalase (CAT), located in peroxisomes, facilitates the breakdown of H_2_O_2_ into water and oxygen. Alternatively, H_2_O_2_ can be transformed (reduced) to H_2_O by glutathione peroxidase (GPx) at the expense of reduced glutathione (GSH), which is oxidized to GSSG. Peroxiredoxins (Prxs) are an unusual family of peroxidases that possess a specific binding site for H_2_O_2_ and play an essential role in the detoxification of hydrogen peroxide. Moreover, peroxiredoxins can be regenerated by the thioredoxin (Trx) redox system, which operates through a specific mechanism. The reduced form of Trx facilitates the reduction of disulfide bonds (S‒S) in oxidized cellular proteins, including peroxiredoxin (Prx). During this reaction, Trx is oxidized and subsequently regenerated by thioredoxin reductase (TrxR) via the use of NADPH as a reducing agent. (Karlenius And Tonissen 2010)

Recent studies have proposed that ROS produced from different and distant sources may “interact” with each other to regulate certain physiological processes (Fukai And Ushio-Fukai 2020). ROS originating from mitochondria and NADPH oxidase may promote angiogenesis, a process involving the formation of new vessels that are essential for embryonic development, peripheral arterial disease, wound healing, tissue repair, and the treatment of various diseases. However, the “mechanism” of how diffusible ROS communicate remains unclear. The crosstalk between mitochondria and NOX has been termed “ROS-induced ROS release,” which positively affects ROS production at specific cell compartments and consequently triggers beneficial redox signaling to modulate cellular processes. A deeper understanding of the mechanisms of ROS interactions originating from different sources, such as mitochondria and NADPH oxidase, may open new avenues for therapeutic interventions in angiogenesis-related cancer and other chronic diseases. Some sources of ROS formation and transformation are schematically outlined in Fig. 1.

### Xanthine oxidase

Xanthine oxidase (XO) is a flavin-containing isoenzyme of xanthine oxidoreductase (XOR) that catalyzes the oxidation of hypoxanthine to xanthine and, in the next step, xanthine to uric acid (Burrage et al. 2023). Activated XO is a physiological source of ROS and RNS, including O_2_^•—^, H_2_O_2,_ and NO^•^. The formation of these radical species may induce a proinflammatory state, predominantly in vascular endothelial cells (Tanaka And Node 2022). XO is gradually upregulated throughout the lifespan because of increased oxidative stress, inflammatory conditions, and other disorders.

### Peroxisomes

In addition to mitochondrial metabolism and the activity of the transmembrane family of NADPH oxidases, another source of endogenous ROS is peroxisomes, which consume large amounts of oxygen and are engaged in ROS generation and scavenging processes. Peroxisomes are small organelles containing more than 50 different enzymes involved in many biochemical processes (Islinger et al. 2018). In this context, catalase is important, because it maintains the physiological level of hydrogen peroxide produced by this organelle from oxygen as a cosubstrate for oxidative purposes. In addition to amino acids, peroxisomes breakdown long-chain fatty acids by oxidation reactions, thus providing a significant source of metabolic energy. Dysfunction of peroxisomes is related to metabolic disorders, and peroxisomes are currently considered protective organelles with a significant impact on human diseases, such as cancer and other chronic diseases, including neurodegenerative and cardiovascular diseases (Poprac et al. 2017; Islinger et al. 2018; Wang et al. 2024).

### Cytochrome P450

The cytochrome P450 (p450 or CYP) superfamily of enzymes is found in the mitochondria and the endoplasmic reticulum of hepatocytes. The active site of cytochrome P450 contains a heme center. The main function of P450 is to metabolize xenobiotics, steroids, fatty acids, and potentially toxic substances and synthesize endogenous molecules. P450 enzymes predominantly function as monooxygenases. The typical reaction catalyzed by P450 is a monooxygenase reaction characterized by the transfer of one oxygen atom of molecular oxygen (O_2_) to an organic substrate (RH) and the reduction of the second oxygen atom to water. The equation gives a simplified reaction mechanism describing the function of P450 (Veith And Moorthy 2018):

3 $$\mathrm{RH}+{\mathrm{H}}^{+}+{\mathrm{O}}_{2}+\text{NADPH }\stackrel{\mathrm{CYP}}{\to }\text{ ROH}+ {\mathrm{H}}_{2}\mathrm{O}+ {\mathrm{NADP}}^{+}$$

Cytochrome P450 reductase is an enzyme that facilitates the transfer of electrons from NADPH to electron acceptors, including P450 and cytochrome c. This enzyme plays a crucial role in the reduction of xenobiotics and other substances across different tissues.

### Endoplasmic reticulum

The endoplasmic reticulum (ER) is a complex membrane-bound subcompartment (rough and smooth) of mutually interconnected tubules and flattened cisterns found in the cytoplasm of most eukaryotic cells (Cooper 2000). The ER is connected to the cell nucleus and usually to the Golgi apparatus. The ER produces many hormones, cytokines, and other proteins that are secreted into the extracellular space. The ER membrane-associated protein endoplasmic reticulum oxidoreductin-1 (EOR1) oxidizes protein disulfide isomerase (PDI), one of the most abundant ER proteins that triggers ROS formation and consequently ER stress (Ochoa et al. 2018). It has been estimated that the oxidation process produces a large proportion of sulfur-derived ROS originating from disulfide bonds.

Calcium release by the ER can also activate ROS formation (Görlach et al. 2015). This is achieved when the ER Ca^2+^-channel, the ryanodine receptor (RyR), is activated by the oxidation of its thiol (-SH) sites (Denniss et al. 2018). Disturbances in calcium homeostasis are associated with mitochondrial dysfunction and increased activity of NADPH oxidase, an important source of ROS (Kovac et al. 2015).

### Nitric oxide synthase (NOS) uncoupling

Nitric oxide (NO^•^) is an important radical species released by endothelial cells. NO^•^ is a signaling molecule that can freely diffuse across cell membranes, modulate various physiological functions, promote blood flow, and promote muscle contractility, primarily in the cardiovascular and nervous systems (Förstermann And Sessa 2012). NO^•^ is generated by three different isoforms of the enzyme nitric oxide synthase (NOS), namely, inducible iNOS, endothelial eNOS, and neuronal nNOS.

Vascular NO^•^ is synthesized by eNOS from L-arginine, with tetrahydrobiopterin (BH_4_) as an essential cofactor. Various pathological states of an organism and aging may affect the activity of eNOS and lead to eNOS uncoupling (Janaszak-Jasiecka et al. 2023). This may increase the level of oxidative stress, which can be one of the key causes of several pathologies, including endothelial dysfunction. Uncoupled eNOS in cells can result in the generation of both O_2_^•—^ and NO^•^ in close proximity. NO^•^ can, via a diffusion-controlled reaction, rapidly react with O_2_^•—^producing the short-lived oxidant peroxynitrite (ONOO^—^) (Jomova et al. 2023):

4 $$O_{2}^{ \bullet - } + NO ^{ \bullet } \to ONOO^{ - }k = \left( {4 - 6} \right)x10^{9} M^{ - 1} s^{ - 1}$$

ONOO^—^ is a very reactive intermediate that can oxidize glutathione (GSH) and damage polyunsaturated fatty acids (PUFAs) and important biomolecules, including DNA. ROS-induced oxidation of PUFAs results in peroxidation of lipids, whose breakdown products, such as 4-hydroxy-2-nonenal (4-HNE), act as signaling molecules triggering inflammation, ferroptosis, inflammation, or other processes.

## Direct antioxidant defense

Cells contain several lines of antioxidant defense against the accumulation and deleterious effects of ROS/RNS (Jomova et al. 2024). The first line of defense involves a group of antioxidant enzymes that play a key role in maintaining redox homeostasis and in coping with oxidative stress.

### Superoxide dismutase (SOD)

Superoxide dismutases (SODs) are among the most important types of O_2_^•—^converting groups of antioxidant enzymes representing the first line of antioxidant defense against oxidative stress. SOD occurs in three different forms, cytosolic Cu, Zn-SOD (SOD1), mitochondrial Mn-SOD (SOD2), and extracellular SOD (EC-SOD, SOD3), providing antioxidant protection in the extracellular space. Cu, Zn-SOD (SOD1) exists as a stable homodimer, with each subunit comprising one copper atom and one zinc atom. Mn-SOD (SOD2) is a homotetramer containing a manganese atom in the active site and has been reported to play a dual role in cancer (Gill et al. 2016). EC-SOD (SOD3) is predominantly found in the lungs and is located primarily in the extracellular space.

The copper atom in SODs is essential for facilitating electron-transfer reactions. SOD enzymes facilitate the dismutation of superoxide anion radical (O_2_^•—^), resulting in the production of hydrogen peroxide (H_2_O_2_), a nonradical ROS. The process of catalytic dismutation of the superoxide radical (O_2_^•—^) is complex and occurs through two diffusion-controlled reactions (Perry et al. 2010). For more details, we refer readers to recent comprehensive reviews (Wang et al. 2018) and references therein.

Nickel superoxide dismutase (Ni-SOD) represents a distinctive category of SOD, notable for its utilization of a nickel cofactor and its unique structural configuration (Barondeau et al. 2004). Ni-SOD is made up of six identical subunits, each of which contains a nickel ion. These subunits assemble to create a homohexamer. Ni-SOD adopts a globular form that resembles a hollow sphere. Each of six subunits is structured as a four-helix bundle, with the helices arranged in an alternating up-down pattern. Ni-SOD is particularly distinctive among SODs for employing cysteine residues (Cys2 and Cys6) to coordinate with the nickel ion at its active site.

#### SOD and cancer

SOD1 plays a multifaceted role in signaling pathways, proliferation, and survival of cancer cells. Elevated levels of SOD1 have been observed in nasopharyngeal carcinoma (NPC), non-small cell lung cancer (NSCLC), and breast cancer (Li et al. 2020a, 2020b). Inhibition of SOD1 by the potent inhibitor ATN-224 (bis (choline)tetrathiomolybdate) increases intracellular O_2_^•−^ levels, which unexpectedly results in the suppression of glutathione peroxidase (GPx) activity, leading to a significantly increased concentration of intracellular H_2_O_2_. H_2_O_2_ is known to promote the expression of proapoptotic factors, such as Bcl-2 interacting mediator (BIM) of cell death and Bcl-2 binding component 3 (PUMA), as well as the phosphorylation of p38MAPK, which subsequently reduces the expression of the antiapoptotic factor myeloid cell leukemia 1 (MCL1) (Parascandolo And Laukkanen 2019). Cancer cells treated with ATN-224 undergo programmed caspase-mediated apoptosis both in vitro and in vivo, indicating that SOD1 may act as a tumor promoter and represents a promising novel target for cancer treatment (Glasauer et al. 2014; Juarez et al. 2008).

Initial studies on the role of SOD2 in carcinogenesis revealed a link between suppressed levels of SOD2 expression during the early stages of cancer development (Oberley And Buettner 1979; Plymate et al. 2003). Conversely, subsequent research revealed that SOD2 expression tends to increase in advanced, aggressive, and metastatic cancers, as well as in various cell models, highlighting the importance of this enzyme in cancer progression. This conclusion is supported by findings demonstrating the role of SOD2-mediated inhibition of apoptosis across multiple cancer types and cell lines, including fibrosarcoma, bladder and breast cancer, and metastatic bladder cancer (Nelson et al. 2003; Hempel et al. 2009).

Recent studies revealed that EC-SOD may exert a tumor-suppressive influence on cancer, although the precise mechanisms remain inadequately elucidated (Griess et al. 2017). Furthermore, low levels of EC-SOD in cancer patients have been associated with unfavorable prognoses, whereas increased EC-SOD expression has been linked to the suppression of tumor growth and metastasis.

### Catalase

Another very efficient enzyme working in tandem with SODs is catalase, the “oldest” enzyme with the highest turnover of all enzymes. In the following reaction, catalase decomposes hydrogen peroxide into water and molecular oxygen (Glorieux And Calderon 2017):

5 $$2{\mathrm{H}}_{2}{\mathrm{O}}_{2} \stackrel{Catalase}{\to } {{2\mathrm{H}}_{2}\mathrm{O}+\text{ O}}_{2}$$

Catalase prevents the excessive formation of hydrogen peroxide, which may be converted to damaging hydroxyl radicals by the Fe-catalyzed Fenton reaction (reaction 2 above).

#### Catalase and cancer

Similar to that of SOD, altered catalase expression has been observed in various tumors compared with normal counterparts; however, the results regarding the level of catalase in tumors are ambiguous. Some studies reported increased levels of catalase, whereas others reported downregulation of this enzyme. Increased levels of catalase expression have been observed in skin cancer, gastric adenocarcinoma, and colorectal cancer (Sander et al. 2003; Hwang et al. 2007; Rainis et al. 2007). Conversely, decreased levels of catalase have been reported in prostatic adenocarcinoma (Baker et al. 1997), human colon carcinoma (Lauer et al. 1999), lung cancer (Chung-man et al. 2001), and pancreatic cancer (Cullen et al. 2003). The decreased levels of catalase indicate increased sensitivity of cancer cells to oxidative stress. Interestingly, cancer cells have been shown to develop resistance to prolonged H_2_O_2_ exposure (Nenoi et al. 2001).

### Glutathione peroxidase (GPx) and peroxiredoxins (Prx)

In addition to catalase, SODs work in conjunction with the glutathione peroxidase family (GPx1–8) of antioxidant enzymes, maintaining the cell redox balance. GPx catalyzes the reduction of H_2_O_2_ (or organic hydroperoxides, ROOHs) to H_2_O (or corresponding alcohols, ROHs) and O_2_ through the oxidation of reduced glutathione (GSH) to its oxidized form (GSSH) (Pei et al. 2023). The following reactions describe the removal of hydrogen peroxide and hydroperoxides catalyzed by GPx:

6 $${\mathrm{H}}_{2}{\mathrm{O}}_{2}+2\text{GSH }\stackrel{GPx}{\to } 2{\mathrm{H}}_{2}\mathrm{O}+\text{GSSG }$$

7 $$\mathrm{ROOH}+2\text{GSH }\stackrel{GPx}{\to }\text{ ROH}+\mathrm{GSSG}+{\mathrm{H}}_{2}\mathrm{O}$$

In addition to catalase and glutathione peroxidases (GPXs), a family of six isoforms of peroxiredoxins (PRDX1–6) also reduce H_2_O_2_ to O_2_. Historically, peroxiredoxins have been divided into three classes: typical 2-Cys PRXs, atypical 2-Cys PRXs, and 1-Cys PRXs. PRXs are highly abundant and, together with catalase, have one of the highest turnovers of all enzymes, making them physiologically unusually significant (Brigelius-Flohé and Maiorino 2013). Interestingly, while PRDX1–5 reduces H_2_O_2_ at the expense of small redox proteins, such as the thioredoxin (TRX) system, PRDX6 preferentially uses glutathione (GSH). Glutathione peroxidases and peroxiredoxins are known to scavenge the most important RNS, nitric oxide (NO^•^), and reduce peroxynitrite (ONOO^—^) (Benhar 2018).

The antioxidant defense grid is complemented by thioredoxin (Trx), a key antioxidant that regulates the dithiol [(R-SH)_2_, reduced form]–disulfide (R–S–S–R, oxidized form) balance of interacting proteins (Samoylenko et al. 2013).

#### Glutathione peroxidase (GPx) and peroxiredoxins (Prx), and cancer

Glutathione peroxidases play a dichotomous role in cancer. Glutathione peroxidase 1 (GPx1) is significantly increased in various cancer types and has complex dichotomous functions as both a tumor suppressor and a promoter, depending on the specific type of cancer (Lee et al. 2017). Gene polymorphisms are associated with susceptibility to various diseases, particularly cancers (Imyanitov et al. 2004). The human GPx1 gene is associated with multiple genetic polymorphisms across various cancers, including breast, bladder, prostate, lung, leukemia, and colon cancers. Despite extensive research, the relationship between these genetic variants and cancer susceptibility remains contentious and ambiguous, largely attributable to variations in study populations and the statistical methodologies employed (Zhao et al. 2022).

A comprehensive analysis revealed that GPx1 is highly expressed in most cancers. These include glioblastoma multiforme, renal papillary cell carcinoma (KIRP), acute myeloid leukemia (AML), low-grade glioma (LGG), ovarian serous cystadenocarcinoma, pancreatic adenocarcinoma, skin melanoma, testicular germ cell tumors, thyroid cancer, and endometrial cancer (Wei et al. 2020).

Glutathione peroxidase 4 (GPx4) functions as a key regulator of ferroptosis, a form of cell death distinct from apoptosis. GPx4 has been found to be elevated in hepatocellular carcinoma and colorectal carcinoma, whereas its expression is notably reduced in breast cancer and renal cell carcinoma compared with normal tissue (Sugezawa et al. 2022). The expression levels of GPX4 in lung cancer, esophageal cancer, and B-cell lymphoma have been correlated with good patient prognosis, highlighting its potential role as a prognostic biomarker (Chen et al. 2024a, 2024b).

**Fig. 2:**
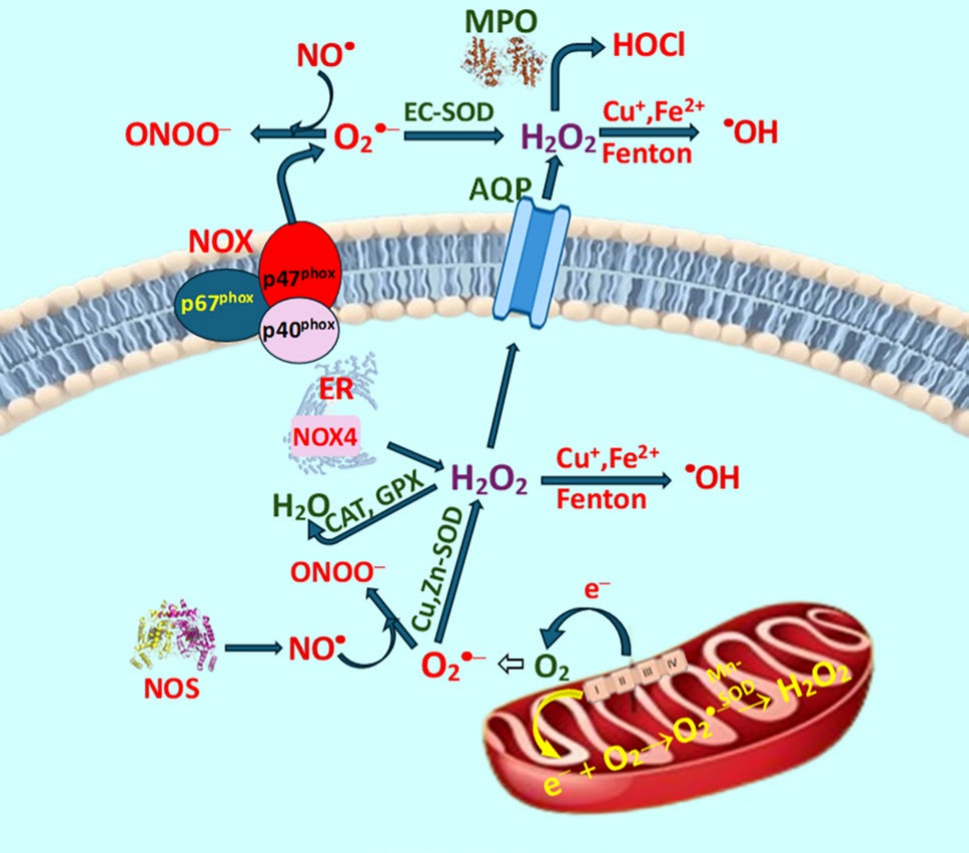
Simplified survey of ROS formation and elimination mechanisms. Abbreviations: Superoxide dismutases (SOD), manganese-SOD (Mn-SOD, SOD2), copper, zinc-SOD (Cu,Zn-SOD, SOD1), extracellular SOD (EC-SOD, SOD3), nitric oxide synthase (NOS), Fenton (Fenton reaction), myeloperoxidase (MPO), catalase (CAT), glutathione peroxidase (GPx), endoplasmic reticulum (ER), NADPH oxidases (NOXs), transmembrane channel proteins—aquaporins (AQP), nitric oxide (NO^•^), peroxynitrite (ONOO^—^), superoxide radical anion (O_2_^•—^), hydroxyl radical (^**•**^OH), hydrochloric acid (HClO). For more details, see Sarmiento-Salinas et al. (2021)

Peroxiredoxins play a protective role in healthy cells against oxidative damage under physiological conditions. In cancer, elevated expression of peroxiredoxins has been linked to increased tumor proliferation and enhanced resistance to radiotherapy (Forshaw et al. 2019). An examination of the expression of peroxiredoxins (PRXs) in human tissues, cell cultures, and animal models revealed that Prx levels are elevated in numerous types of cancer, as evidenced by both mRNA and protein analyses. In a significantly smaller number of cases, the PRX level was found to be reduced (Hampton et al. 2018). In addition, conditions such as hypoxia and inflammation are likely to exacerbate this phenomenon. The mechanisms of ROS formation and elimination are outlined in Fig. 2.

### Low-molecular-weight antioxidants and cancer

The most efficient antioxidants, such as glutathione, are those synthesized in the human body. Nevertheless, certain dietary compounds that exhibit antioxidant properties in vitro include ascorbate, vitamin E, flavonoids, and carotenoids. The beneficial in vivo antioxidant effects of all these antioxidants, except vitamin E, remain limited, as documented by various clinical trials (Halliwell 2022).

In contrast to detailed knowledge about the distribution of antioxidant enzymes, there is a notable lack of information regarding the cellular and tissue localization of low-molecular-weight antioxidants, including ascorbate, tocopherol, carotenoids, flavonoids, and other low-molecular-weight antioxidants (Jomova et al. 2024). The effective therapeutic use of low-molecular-weight antioxidants necessitates a multifaceted strategy. It is essential to consider various factors, including the amount and site of their location, the structural characteristics of antioxidants, and the age and health condition of the individual receiving the treatment.

#### Glutathione

Glutathione is an intracellularly synthesized antioxidant that protects cells from oxidative damage and maintains redox homeostasis. In most cells, GSH is present at millimolar concentrations; hepatocytes contain glutathione at significantly higher concentrations (~ 10 mM) (Forman et al. 2009). GSH has been found to be increased in various cancer cells (Kennedy et al. 2020).

#### Vitamin C (ascorbic acid)

Vitamin C is a water-soluble vitamin that functions as an electron donor or reducing agent and serves as a cofactor for several enzymes in mammals (Nauman et al. 2018a, 2018b). Ascorbic acid exerts its physiological functions primarily through the transfer of electrons from ascorbate to a target molecule. At physiological pH, ascorbic acid is present in the form of an ascorbate anion (AscH^—^). Reactions of ascorbate with radicals (R^•^) result in the formation of ascorbyl radicals (Asc^•—^) according to the following reaction:

8 $$AscH^{ - } + R^{ \bullet } \to Asc^{ \bullet - } + RH$$

The accumulation of ascorbic acid varies across different tissues and bodily fluids. The electrons originating from ascorbate can also reduce metals, such as copper and iron, which may result in the production of superoxide and hydrogen peroxide, ultimately leading to the formation of reactive oxidant species.

#### Vitamin E (α-tocopherol)

Vitamin E is classified as a fat-soluble vitamin that exists in various forms, with alpha-tocopherol being the sole variant utilized by the human body (Krinsky et al. 2003). Its primary function is to serve as a powerful chain-breaking antioxidant, effectively preventing ROS formation during free radical reactions and the oxidation of fats. The results of clinical research examining the prevention of various chronic diseases, including cancer, do not provide evidence in favor of the preventive benefits of vitamin E supplementation. Vitamin E can be regenerated from its α-tocopheroxyl radical form (α-TO^•^) to α-tocopherol (Jomova et al. 2024).

#### Carotenoids

Carotenoids are color pigments that occur naturally in various organisms, including plants, bacteria, fungi, and algae (Jomova et al. 2013). These pigments play numerous roles, particularly because of their ability to scavenge ROS, interact with light, and protect against photodamage. The major factors affecting the antioxidant properties of carotenoids are the carotenoid concentration at the site of action, the redox potential (0.63–0.94 V), the partial pressure (concentration) of oxygen at the site of action, the presence of redox-metal ions and other factors (Focsan et al. 2021; Jomova et al. 2022). Therefore, carotenoids may undergo a switch from antioxidant to prooxidant properties depending on the conditions under which they work. This was documented by a well-known large-scale randomized placebo-controlled trial examining the impact of supplemental β-carotene (ATBC 1994). The outcome of this trial revealed significant prooxidant properties of beta-carotene, increasing the incidence of lung cancer (see below).

#### Flavonoids

**Fig. 3:**
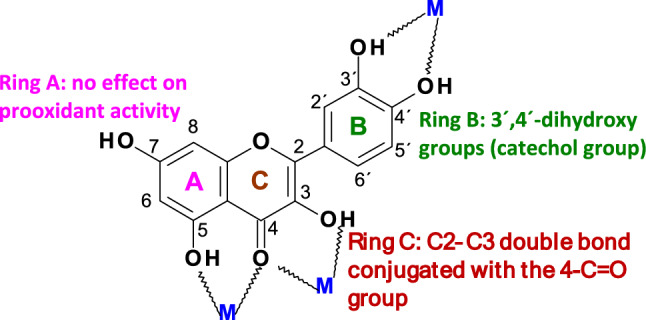
Basic skeleton of flavonoids (M = coordinated metal ion)

Flavonoids represent a diverse group of polyphenolic compounds that play significant roles in various physiological processes within plants (Simunkova et al. 2019). The antioxidant/prooxidant characteristics of flavonoids are influenced by the number and position of hydroxyl groups, which is supported by their ability to chelate redox-active metal ions, including iron and copper (Jomova et al. 2025) (Fig. 3). In addition, the planarity of the molecular framework and the presence of a conjugated π-bond system extended to the carbonyl group due to the presence of the C2‒C3 double bond impact the antioxidant efficiency of flavonoids. Quercetin and other flavonoids from different subgroups have been shown to exhibit inhibitory effects in various animal models of cancer, including those affecting the lung, colon, and prostate (Jomova et al. 2025). However, the findings from observational studies remain inconclusive (Romagnolo And Selmin 2012).

Taken together, the evidence supporting the efficacy of these compounds under in vivo conditions, particularly in humans, is limited. Clinical trials investigating the effects of small-molecule antioxidants on the prevention or treatment of chronic diseases in humans have largely yielded unsatisfactory outcomes (Halliwell 2022). One of the main reasons for this is that these high doses of vitamin supplements are generally ineffective at reducing the rate of oxidative damage in the human body.

## Indirect antioxidant defense

In addition, cells have indirect-acting antioxidant mechanisms that either detoxify the reactive metabolites they produce or restrict the production of ROS/RNS.

### Xenobiotic metabolism in antioxidant defense

Xenobiotic-metabolizing enzymes typically facilitate detoxification. Phase I is dominated by cytochrome P450s (CYPs). Particularly important for the metabolism of xenobiotics are the CYP1, CYP2, CYP3, and CYP4 families, which are activated by barbiturates, glucocorticoids, polycyclic aromatic hydrocarbons, and peroxisome proliferators (Stanley et al. 2017). Excretable hydrophilic metabolites are usually produced during phase II metabolism by glutathione S-transferases, uridine 5′-diphospho (UDP)-glucuronosyltransferases, sulfotransferases, N-acetyltransferases, and epoxide hydrolases. These systems may prevent quinones/hydroquinones from redox cycling reactions, the formation of lipid peroxidation products, GSH depletion, and the inactivation of signaling molecules such as hydroxynonenal and other molecules derived from the lipid peroxidation process capable of triggering apoptosis and ferroptosis (Dodson et al. 2019).

### Sirtuin 3 as a regulator of oxidative stress

Sirtuin 3 is an NAD^+^-dependent deacetylase that plays a role in mitochondrial metabolic processes, including energy production, oxidative stress, the tricarboxylic acid cycle, and other processes (Bause et al. 2013).

Owing to its ability to deacetylate substrates implicated in ROS generation and detoxification, sirtuin3 is recognized as a critical regulator of oxidative stress. Acetylated mitochondrial SOD2 results in decreased dismutase activity and acquired peroxidase activity, which promotes oxidative damage and sensitizes cells to peroxide stress (Hjelmeland et al. 2019). Sirtuin 3 has the capacity to catalyze the deacetylation of mitochondrial SOD2, which in turn increases the scavenging efficiency of O_2_^•—^ in mitochondria. Sirtuin 3 can also catalyze the deacetylation of the mitochondrial enzyme isocitrate dehydrogenase-2 (IDH2), which promotes mitochondrial NADPH production (Kincaid And Bossy-Wetzel 2013).

### Iron-based proteins and heme oxygenase as antioxidants

Redox-metal sequestering proteins such as ferritin, ferroportin, ceruloplasmin, and other proteins play crucial roles in maintaining redox homeostasis and preventing the formation of ROS, for example, through the redox-metal-catalyzed Fenton reaction, which damages hydroxyl radicals (^•^OH). Ferritin is a globular protein complex that oxidizes and stores iron (up to 4500 Fe^3+^ ions) (Kotla et al. 2022). Ferroportin is a transmembrane protein that transports iron from the intracellular space to the extracellular environment via the iron chaperone poly (RC)-binding protein 2 (PCBP2), which has been found to have ambiguous functions in a variety of cancers (Vlasveld et al. 2019). Ceruloplasmin (Cp) is a Cu-containing ferroxidase that exhibits antioxidant functions in part by oxidizing toxic Fe^2+^ to nontoxic Fe^3+^ iron, thus maintaining iron homeostasis. Ceruloplasmin is also responsible for blood copper transport (Chen et al. 2022).

## Compartmentalization of ROS production and antioxidant defense

Nature has developed a system of cellular ROS/antioxidant compartmentalization mechanisms that ensure that redox homeostasis is maintained by the activity of ROS-producing/degrading systems. NADPH oxidases (NOXs) act at distinct subcellular locations, including the endoplasmic reticulum, mitochondria, peroxisomes, and plasma membrane. Although NADPH is unable to translocate to the mitochondria across the inner membrane, its exchange between the cytosol and mitochondria is achieved by NADPH shuttle functions through the enzymes isocitrate dehydrogenase 1, [NADP +] (IDH1), and isocitrate dehydrogenase [NADP] mitochondrial (IDH2) (White et al. 2023).

Mitochondrial trafficking can fine-tune ROS production in different subcellular compartments and affect distant ROS-mediated signaling, which has been implicated, for example, in regulating repair pathways (Horn et al. 2020). In response to various stimuli, ROS-regulating systems might enable a targeted and localized response. These involve, for example, the redistribution of mitochondria to cell compartments lacking energy for cellular processes (Caino et al. 2015) or localized ROS production by NOXs at sheet-like membrane protrusions (lamellipodia) of neurons and immune, epithelial, and other cells (Gianni et al. 2009).

In addition to ROS-regulating enzymes, families of antioxidant enzymes such as peroxiredoxins (PRXs), glutathione peroxidases (GPXs), and glutathione S-transferases (GSTs) are also present in different subcellular locations and organelles, including peroxisomes and mitochondria, primarily because these organelles contain a number of ROS-regulating/producing enzymes (Samoylenko et al. 2013). As discussed above, SOD occurs in three different forms: Cu, Zn-SOD (SOD1) is localized primarily in the cytosol; Mn-SOD (SOD2) is found mostly in the mitochondria; and extracellular SOD (SOD3) provides antioxidant protection in the extracellular space. The H_2_O_2_-converting enzyme catalase, which works in tandem with SODs, is found primarily in peroxisomes, chloroplasts, and the cytosol. Therefore, the reduction of H_2_O_2_ in mitochondria is carried out predominantly by GPx. The enzymes PRX1, 2, 4, 5, and TRX1 and the low-molecular-weight antioxidants vitamin C and GSH work predominantly in the cytosol. In addition to Mn-SOD, mitochondria are organelles, where GPx1, GPx4, PRX1, PRX3, and vitamins C, E, and the antioxidant GSH work in concert. The plasma membrane is protected against ROS-induced damage by vitamin E, carotenoids, and polyphenols anchored into the membrane structures.

## Cell signaling in the control of redox homeostasis and cancer

Redox-sensitive signaling pathways employ ROS to convey signals from the cell membrane to the nucleus, thereby regulating cellular redox homeostasis (Powers et al. 2011). The transcription factors activated by ROS belong to different families and include activator protein (AP-1), nuclear factor kappa B (NF-κB), nuclear factor erythroid 2-related factor 2 (Nrf2), hypoxia-inducible factor 1 subunit alpha (HIF-1α), and tumor suppressor protein p53 (Tp53). The activity of all these transcription factors, together with the forkhead box class O (FoxO) family of multifunctional transcription factors, regulates redox status and is implicated in the mechanism of carcinogenesis (Brown And Webb 2018).

Nrf2 represents the first line of defense and cellular response to oxidative stress and is activated by a mild increase in the level of ROS/RNS. The second line of defense, which usually responds to significantly increased levels of oxidative stress, is represented by AP-1 and NF-κB; the ultimate level is the activation of apoptosis (Xiao et al. 2003).

The relationship between AP-1 and NF-κB has not been convincingly demonstrated. The “intermediate” levels of ROS trigger, among other factors, FOXO and HIF-1α. TP53 most notably induces cell cycle arrest and cell death or apoptosis and represents the ultimate response to excessive levels of ROS (Hayes et al. 2020).

The degree to which individual components of the aforementioned network of transcription factors are activated in response to oxidative stress remains unclear; however, it is unlikely that all these factors are activated concurrently (Cheung And Vousden 2022). Instead, various transcription factors more likely respond to certain threshold levels of ROS and RNS in a manner that is dependent on both concentration and duration and is likely influenced by the presence of inflammation, DNA damage, metabolic stress, and other factors.

### Nuclear factor kappa B (NF-κB)

NF-κB is a family of transcription factors comprising five monomers, p50, p52, p65/RelA, RelB, and c-Rel that act as the first-line defense against infections and harmful pathogens (Perkins 2007). This is achieved by the upregulated expression of proinflammatory cytokines, chemokines, and receptors and the modulated expression of antioxidant genes (Morgan and Liu 2011). A considerable proportion of human cancers exhibit persistent NF-κB activity, which can be attributed to the inflammatory microenvironment and a range of oncogenic mutations. The activity of NF-κB is associated with increased expression of antiapoptotic genes, increased proliferation of tumor cells, inhibition of apoptosis, and promotion of angiogenesis (Perkins 2012). In addition, NF-κB plays a crucial role in inducing epithelial‒mesenchymal transition, thereby enhancing the potential for distant metastasis (Xia et al. 2014). In addition, NF-κB promotes metabolic transformation and regulates the protumorigenic behavior of immune cells, thus affecting the tumor microenvironment (Taniguchi And Karin 2018).

### Activator protein-1 (AP-1)

AP-1 is a downstream target of the MAPK signaling cascade. The AP-1 family of transcription factors includes various combinations of proteins, such as Jun, Fos, ATF, MAF, and JDP (Verma et al. 1990). These factors induce genes important for antioxidant activities, such as GSH synthesis, maintaining the labile iron pool within physiological limits, ROS scavenging, and xenobiotic metabolism (Glorieux et al. 2016).

AP-1 activity encompasses various cellular processes, including proliferation, migration, and invasion. Abnormalities in AP-1 activity are linked to the onset, progression, invasion, migration, and resistance to treatment of cancer. AP1 has dual functions; it can act as an antioncogenic factor through the induction of apoptosis while simultaneously exhibiting oncogenic properties via a variety of signaling mechanisms (Eferl And Wagner 2003).

Numerous studies have demonstrated the significant involvement of AP-1 components in the process of oncogenesis (Gazon et al. 2018). The genes c-jun and c-fos were initially recognized as retrovirus-activated genes with oncogenic capabilities (Abate And Curran 1990). Exposure to carcinogenic agents can trigger carcinogenic mechanisms by activating a diverse range of signaling pathways, including inflammatory, proproliferative, and survival pathways. A variety of human cancers overexpress members of the Jun family (Kharman-Biz et al. 2013). In support of the idea that c-Jun may enhance tumorigenicity, its overexpression has been documented in certain aggressive forms of CD30-positive lymphomas, such as classic Hodgkin lymphoma (cHL) and peripheral T-cell lymphomas (PTCLs) (Drakos et al. 2007). In breast cancer, alterations in the vascular endothelial growth factor (VEGF) and epidermal growth factor receptor (EGFR) signaling pathways have been linked to the overexpression of c-Jun (Kharman-Biz et al. 2013).

### Nuclear factor erythroid 2-related factor 2 (Nrf2)

Nuclear factor erythroid 2-related factor 2 (Nrf2) serves as the principal transcriptional regulator of cellular responses to oxidative/electrophilic stresses (Ngo And Duennwald 2022). It orchestrates the expression of numerous genes associated with antioxidants and detoxifying enzymes. The activity of Nrf2 is repressed by a redox-sensitive Kelch-like ECH-associated protein (Keap1), which acts as a substrate adaptor protein that interacts with Nrf2 in the cytoplasm, promoting its polyubiquitination by the Cullin 3 (Cul3) E3 ubiquitin ligase (McMahon et al. 2003). Under physiological conditions, Nrf2 is continuously degraded, and its expression is maintained at low/physiological levels. Under oxidative stress conditions, modified cysteine residues of Keap1 are responsible for the inhibition of Nrf2 ubiquitination by Cul3 ([Wakabayashi et al. 2004). Consequently, stabilized Nrf2 is translocated to the nucleus, where it forms heterodimers with the essential cofactors small Maf (sMaf) proteins and binds to the antioxidant response element (ARE). This promotes the expression of genes important for the synthesis of enzymes crucial for ROS detoxification (Ngo And Duennwald 2022).

An intriguing aspect of NRF2 is its lack of Cu,Zn-SOD (SOD1), and Mn-SOD (SOD2) regulation, which indicates that it does not affect O_2_^•−^-mediated redox signaling. Various cellular stresses can upregulate NOX4, a significant source of O_2_^•−^, and Nrf2 has been reported to downregulate its expression (Hayes et al. 2020). Although Nrf2 can repress genes responsible for the expression of proinflammatory cytokines, such as interleukin-1β (IL-1β) and interleukin-6 (IL-6), and thus suppress ROS formation, in general, the inhibition of prooxidant genes by Nrf2 has not yet been satisfactorily documented (Kobayashi et al. 2016). The dual functions of Nrf2 include, on one hand, the promotion of late stages of tumorigenesis by Nrf2 and, on the other hand, its protective effects on anticancer chemopreventive agents.

### Tumor protein 53 (Tp53)

The TP53 gene is responsible for encoding the tumor suppressor protein p53, which possesses domains for transcriptional activation, DNA binding, and oligomerization. The primary mechanism by which p53 safeguards against tumorigenesis occurs through the regulation of genes that are critical for cell cycle arrest, senescence, and apoptosis (Bieging et al. 2014). The exact mechanism by which ROS activate p53 remains ambiguous, as it is uncertain whether this activation is mediated by DNA damage, redox signaling, or a combination of both pathways. However, the most abundant signaling molecule, H_2_O_2_, triggers signaling cascades, including JNK/p38MAPK, which can activate p53 independently of the DNA damage response (Shi And Dansen 2020a 2020b; Nguyen et al., 2018). Under physiological conditions of oxidative stress, p53 functions predominantly as an antioxidant, protecting cells against oxidative damage. Conversely, an increased level of oxidative stress triggers the activity of p53 to exacerbate the level of oxidative stress, ultimately resulting in cell death (Liu et al. 2020a, 2020b).

P53-induced activation of antioxidant genes enhances the cellular antioxidant capacity, which is substantiated by increased synthesis of reduced glutathione (GSH), production of NADPH, and detoxification of electrophiles and oxidants; conversely, it represses the expression of prooxidant enzymes, such as cyclooxygenases (COXs), lipooxygenases (LOXs), xanthine oxidase (XO) and other prooxidant enzymes (Maillet And Pervaiz 2012). Additional prooxidant activities associated with the upregulation of p53 include p53-inducible gene 3 (PIG3).

The expression of p53 inducible gene 3 (PIG3) is modulated by the tumor suppressor p53. PIG3 acts as a quinone oxidoreductase/zeta-crystallin that generates ROS through the redox cycling reactions of quinones. Its activation is influenced by mutations and post-translational modifications of p53. While p53 mutations are common in various tumors, the regulatory impact of different p53 mutants on PIG3 is not straightforward. It has been reported that the expression levels of both PIG3 mRNA and protein are significantly elevated in non-small cell lung cancer (NSCLC) patients with lymph node metastasis compared with those without such metastasis (Gu et al. 2018). PIG3 knockdown significantly inhibited the migration and invasion ability of NSCLC cells and decreased paxillin, phospho-focal adhesion kinase (FAK), and phospho-Src kinase expression, while its overexpression resulted in the opposite effects.

### Hypoxia-inducible factor 1alpha (HIF1α)

HIF1/Arnt is a heterodimer complex that is composed of the alpha subunit (HIF1α) and the beta subunit, aryl hydrocarbon receptor nuclear translocator (Arnt) (Semenza 2012; Mandl And Depping 2014). The expression of HIF-1α protein sharply increases with decreasing oxygen pressure (Wang et al. 1995). The HIF pathway can protect various cell types through specifically tailoring HIF responses to hypoxic microenvironmental sites (Taylor And Scholz 2022).

Under hypoxic conditions, the level of GSH is reportedly increased, which is correlated with the increase in and stabilization of HIF-1α in glial cells (Badawi et al. 2012). This observation indicates that alterations in the redox state under hypoxia may regulate the expression of HIF-1α.

One of the key adaptive responses to hypoxia, regulated predominantly by hypoxia-inducible factors, is angiogenesis (Monaci et al. 2024). Activated HIF-1α enhances the expression of proangiogenic genes, including vascular endothelial growth factor (VEGF), which participate in the initial stages of tumor development, progression, and metastasis (Semenza et al. 2012; Yang And Cao 2022).

Heat shock transcription factor 1 (HSF1) upregulates genes encoding chaperones, termed heat shock proteins (HSPs), and plays a significant role in tumor progression by regulating HIF-1. HSF1 is sensitive to heat shock and nonheat (e.g., ROS) shock conditions. Two cysteine residues (Cys35 and Cys105), identified in the DNA binding domain of HSF1, directly affect the cellular redox state via the induction of antioxidant genes (Barna et al. 2018).

## Double-edged sword of ROS in cancer

It is well-known that ROS have dual functions in biological systems. At low-to-moderate levels, termed good stress or eustress, ROS maintain redox signaling pathways crucial for the normal physiological function of cells (Azzi 2022). Conversely, at moderate-to-high concentrations, ROS can damage various biomolecules, including proteins, DNA, and membrane lipids. This condition, which arises from a disturbed balance between the production of ROS/oxidants and their removal by antioxidants in favor of oxidants, is known as oxidative stress (Sies 2023). Experimental limitations associated with direct in vivo measurements of ROS (detection limit for ROS using quantitative EPR spin trapping is ~ nM), which typically have short half-lives (seconds to µs), require their quantification through indirect methods, such as DNA damage, membrane lipid peroxidation, and protein modifications.

ROS are involved in all stages of the carcinogenic process. Various mechanisms discussed above, such as altered mitochondrial function, increased hypoxic conditions, oncogene activation, loss of tumor suppressor activity, and altered stromal and immunological interactions, are characterized by increased ROS production. However, ROS act as a double-edged sword in oncogenesis, promoting and suppressing cancer (Cheung And Vousden 2022).

### ROS as protumorigenic species

A variety of ROS, especially their prominent member H_2_O_2_, operating within physiological limits, are central players in redox signaling; however, when upregulated, ROS may act as damaging species capable of reversibly oxidizing the cysteine side chains of proteins (Reczek And Chandel 2017). Advanced analytical techniques detect the reversible oxidation of many proteins involved in cell signaling pathways important for the proliferation and survival of cancer cells. The damaging effect of ROS is manifested by DNA damage and genomic instability, promoting cancer incidence. Some examples are discussed below.

#### ROS signaling in DNA methylation

The genetic material of human cells is organized in chromatin, which may undergo processes of DNA methylation and histone modifications (Zhao et al. 2021). ROS-induced DNA damage may trigger decondensation of the local chromatin architecture, incorporation of histone variants, and abnormal chromatin mobility. Alterations in histone and chromatin dynamics that occur in response to ROS-induced DNA damage are important prerequisites of cancer development (Mohan et al. 2021).

The role of ROS in DNA methylation under physiological and pathological conditions has been extensively studied (Hayes And Knaus 2013). DNA methylation is facilitated by the action of methyltransferase enzymes (DNMTs), which utilize the positively charged intermediate S-adenosyl-L-methionine (SAM) (Afanasev 2013). ROS have been shown to be involved in hypermethylation at specific sites through the upregulation of DNA methyltransferase expression. ROS-induced DNA methylation patterns are associated not only with malignant transformation but also with the progression of various tumors (Wu And Ni 2015).

ROS-mediated DNA methylation results in the silencing of certain tumor suppressor genes, thereby facilitating tumor progression. Hepatocellular carcinoma cells, characterized by increased exposure to ROS, have been shown to induce the methylation of CpG islands (CpG islands refer to genomic regions characterized by a high abundance of CpG dinucleotides relative to other areas of the genome) within the cadherin promoter (Lim et al. 2008). In addition, hypermethylation of the p16 gene promoter contributes to inactivation of the p16 tumor suppressor gene, which is implicated in the progression from Barrett’s esophagus to esophageal adenocarcinoma.

The function of antioxidant enzymes, including catalase, is significantly reduced in hepatocellular cancer. ROS have been found to induce the methylation of CpG island II within the catalase promoter, leading to a decrease in catalase expression at the transcriptional level in hepatocellular cancer (Min et al. 2010). Thus, the progression of hepatocellular cancer may be attributed to the downregulation of the catalase gene by ROS through the methylation of its promoter region.

#### ROS and DNA damage

DNA damage is a significant obstacle to all five stages of mitosis (the process of cell division), involving the synthesis of complementary DNA strands during replication, chromosome segregation, and the final stage of physical cell division, cytokinesis (Groelly et al. 2023). The level of DNA damage and mutations varies significantly across different cell types, tissues, and individuals, shaped by intrinsic differences in genomic maintenance mechanisms and the impact of environmental factors. Hematopoietic stem cells (HSCs) can develop into all types of blood cells. They can undergo numerous cell cycles of self-renewal, proliferation, and differentiation and are particularly susceptible to DNA damage.

**Fig. 4:**
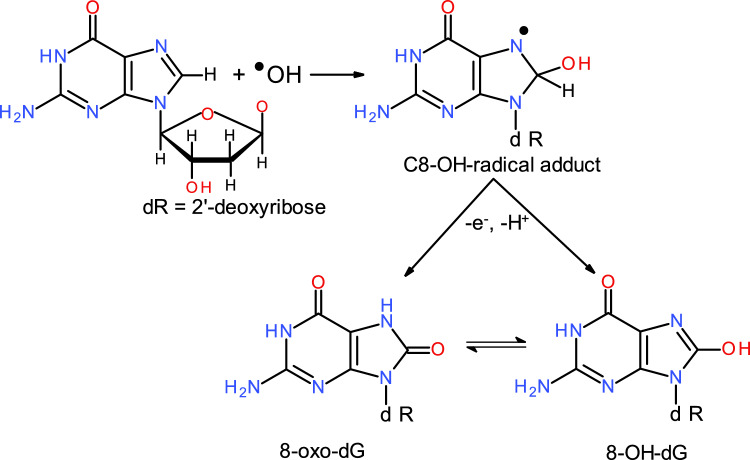
Reaction of 2’-deoxyguanosine with^•^OH results in the formation of a C8-OH radical adduct, which can be oxidized to 8-hydroxy-2’-deoxyguanosine (8-OH-dG) or its tautomeric form 8-oxo-7-hydro2’-deoxyguanosine (8-oxo-dG)

Among the nucleobases, guanine has the lowest oxidation potential; therefore, its susceptibility to oxidation is the highest of all four DNA bases (Huang et al. 2021b; Chepelev et al. 2015). This susceptibility facilitates the formation of 8-oxoguanine in DNA (8-oxo-2'-deoxyguanosine and 8-oxo-dG), which introduces mutagenic lesions resulting in mispairing. 8-oxo-dG is one of the most frequently studied DNA oxidation products and results from the interaction between guanine and the hydroxyl radical (^•^OH), which originates, for example, from the Fenton reaction (see above) (Fig. 4). 8-oxo-dG induces a G > T (C > A) mutation in cancers, which can be deleterious (Hahm et al. 2022; Valavanidis et al. 2009; Kant et al. 2016). Many other oxidative DNA lesions, such as 5-hydroxy-2’-deoxycytidine and 5, 6-dihydro-5, 6-dihydroxy-2’-deoxyuridine, have also been shown to be mutagenic.

ROS can lead to the disruption of hydrogen bonds and cause the unfolding and fragmentation of both double-stranded and single-stranded regions of the DNA double helix. This mechanism increases the vulnerability of purine and pyrimidine residues to ROS attacks, subsequently promoting the oxidation of nucleobases, which plays a significant role in the development of DNA mutations.

#### ROS and immune response

ROS can promote tumorigenesis through the regulation of immune cells (Huang et al. 2021b). The ROS-induced activation of immune cells generally results in antitumor effects; however, overproduction of ROS can result in oxidative stress, negatively impacting immune cell functionality and undermining antitumor immunity. In the context of the tumor microenvironment of solid tumors (the heterogeneous assembly of nonmalignant cells, including tumor-associated fibroblasts, tumor-associated immune cells, bacteria, and vascular cells surrounding cancer cells), ROS can exert immunosuppressive effects predominantly on T cells and natural killer (NK) cells (Kennel And Greten 2021).

T cells are a diverse group of lymphocytes that form an essential part of the adaptive immune system. The most common T cells are CD4 + T cells (helper cells) and CD8 + T cells (killer T cells). ROS can regulate the immune response of T cells. T cells are particularly important in the tumor microenvironment (TME). Since T-cell-intrinsic ROS are implicated in cell activation, differentiation, apoptosis, and antigen recognition, their role in tumor progression is highly anticipated.

The role of adaptive immune responses in the context of nonalcoholic fatty liver disease (NAFLD)-induced hepatocellular carcinoma (HCC) has been investigated (Ma et al. 2016). The dysregulation of lipid metabolism associated with NAFLD results in preferential depletion of intrahepatic CD4 + T lymphocytes, whereas CD8 + T lymphocytes remain unaffected, thereby accelerating the process of hepatocarcinogenesis. In addition, the findings revealed that CD4 + T lymphocytes possess a greater mitochondrial mass than do CD8 + T lymphocytes and produce elevated ROS levels derived from mitochondria. The impairment of mitochondrial function caused by linoleic acid, a fatty acid that accumulates in NAFLD, leads to greater oxidative damage than that caused by other free fatty acids, such as palmitic acid, and contributes to the selective depletion of intrahepatic CD4 + T lymphocytes. The effective antioxidant N-acetyl cysteine (NAC) is able to suppress the growth of tumors.

In a type of kidney cancer, clear cell renal cell carcinoma (ccRCC), CD8 + tumor-infiltrating T cells exhibit signs of inactivation and generate substantial levels of ROS (Siska et al. 2017). The application of Mito-TEMPO, a mitochondrially targeted superoxide and alkyl radical ROS scavenger/SOD mimetic, has been shown to partially restore the functionality of these T cells.

A specialized type of white blood cell of the innate immune system, macrophages play an integral role in the immune system in response to increased levels of ROS. The upregulation of various signaling pathways, such as the MAPK, JAK/STAT, WNT, NF-κB, and PI3K/AKT pathways, promotes the synthesis of immunosuppressive checkpoint inhibitors, such as programmed death-ligand 1 (PD-L1), which are usually expressed by macrophages, some activated T cells and B cells (Dong and Markovic 2018). PD-L1 is a 40 kDa type 1 transmembrane protumorigenic protein in cancer cells and a coinhibitory factor of the immune response. Recent research has confirmed that the expression of PD-L1 is associated with epithelial‒mesenchymal transition (EMT), which is a physiological process in which epithelial cells lose their characteristic properties and are transformed into mesenchymal cells characterized by differences in mobility and invasiveness (Ock et al. 2016). PD-L1 expression has been associated with EMT in human estrogen receptor-positive (ER +) breast cancer cell lines (MCF7) and the estrogen receptor-negative (ER-) breast cancer cell lines BT549 and MDA-MB-231 (Han et al. 2020).

#### ROS and proteins

Ras-related C3 botulinum toxin substrate 1 (Rac1) is an important member of the Rho GTPase family and plays crucial roles in the regulation of cell proliferation, reorganization of the cytoskeleton, and the process of cell migration (Pervaiz And Chong 2018). Rac1 has been shown to activate NOX1, a homolog of the catalytic subunit of the superoxide-generating NADPH oxidase of phagocytes (gp91phox) (Cheng et al. 2006). Rac1-activated NOX1 produces ROS, which have been implicated in the process of lung and skin tumorigenesis (Myant et al. 2013).

Another example of the oncogenic activity of Rac1 was further confirmed by Rac1-triggered ROS production and NF-κB activation, which have been shown to be critical events in wingless-type MMTV integration site family (WNT)-driven initiation of colorectal cancer (Myant et al. 2013).

#### ROS and mitochondria

**Fig. 5:**
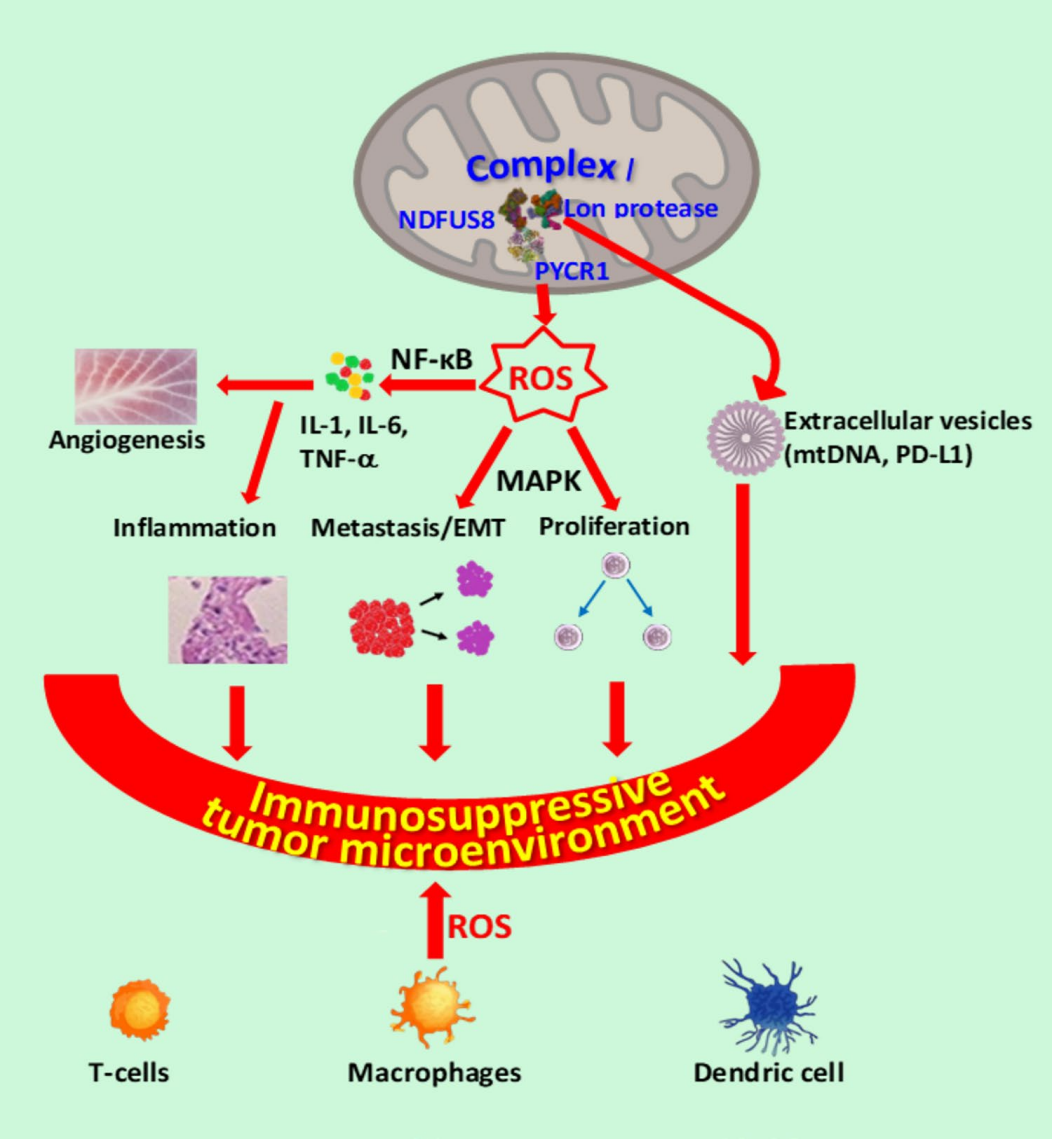
Selected pathways of mitochondrial ROS stress. Abbreviations: NADH Ubiquinone Oxidoreductase Core Subunit S8 (NDFUS8), Pyrroline-5-carboxylate reductase 1 (PYCR1), Lon protease family (Lon), proinflammatory cytokines (IL-1, IL-6, and TNF-α), epithelial–mesenchymal transition (EMT), programmed death-ligand 1 (PD-L1), extracellular vesicles (EVs)

Mitochondria are important sources of ROS (mtROS) in cancerous and normal cells (Fig. 5). Mitochondrial metabolism is essential for cancer cell proliferation, survival, and metastasis. The main source of mtROS originates from the activity of mitochondrial complex I (Kong et al. 2020). mtROS regulate various signaling pathways that enhance signaling, resulting in oncogenic phenotypes. The expression of Rac1b, an isoform of Rac1, has been implicated in cancer development through mtROS-induced cell damage (Radisky et al. 2005).

Kirsten rat sarcoma viral oncogene homologs (Krass) belong to a group of the most commonly mutated oncogenes in cancer, serving as initiators of tumorigenesis and powerful promoters of malignancy (Huang et al. 2021a ). A study of an oncogenic Kras-driven mouse model of lung cancer revealed that mitochondrial ROS production and mitochondrial metabolism are essential factors affecting Kras-induced cell proliferation and tumorigenesis.

Mitochondrial oxidative stress mediates immunosuppression in the tumor microenvironment. It is widely recognized that mitochondrial ROS play a crucial role in the progression of inflammation, significantly affecting the tumor microenvironment (Fig. 5) (Kuo et al. 2022). The overproduction of ROS leads to prolonged inflammation, a key factor contributing to tumor immunosuppression. Lon is a mitochondrial master protease that functions as a chaperone. It is a vital enzyme for maintaining mitochondrial function and DNA integrity. Lon is upregulated in response to ROS and may interact with other mitochondrial proteins, such as NADH Ubiquinone Oxidoreductase Core Subunit S8 (NDFUS8) and/or Pyrroline-5-carboxylate reductase 1 (PYCR1) (Kuo et al. 2020). This interaction boosts mitochondrial ROS production, which influences various cellular processes, including cell proliferation and tumor progression. Lon-mediated ROS production activates signaling pathways essential for cell proliferation, including Ras-ERK(ERK1/2), MAPK(P38) (Cheng et al. 2013; Kuo et al. 2020), and WNT (β-catenin) (Gibellini et al. 2018). To escape oxidative stress in the tumor microenvironment, Lon-induced ROS can activate the MAPK or NF-κB pathways, thereby enhancing cell migration and invasion. ROS stimulate cancer cells to secrete NF-κB-dependent inflammatory cytokines, including VEGF, IFN-γ, TGFβ, IL-6, IL-4, and IL-10, which contribute to the establishment of an immunosuppressive microenvironment affecting regulatory T cells (Treg), macrophages, and dendritic cells (DC) (Cheng et al. 2013). In addition, hypoxia and ROS-induced upregulation of Lon promote the release of extracellular vesicles (EVs) containing mitochondrial DNA (mtDNA) and programmed death-ligand 1 (PD-L1). These EVs, produced in response to mitochondrial ROS (mtROS), further enhance the secretion of IFN and IL-6 from macrophages, thereby suppressing T-cell-mediated immunity within the tumor microenvironment (TME).

#### Sirtuin 3

The role of Sirtuin 3 (SIRT3) in cancer is very intriguing. SIRT3 is a prominent mitochondrial NAD + -dependent deacetylase known to govern mitochondrial metabolic pathways and mediate interactions between mitochondria and intracellular signaling (Ouyang et al. 2022).

The use of various cancer models to reveal the specific function of SIRT3 in tumorigenesis has yielded ambiguous conclusions. SIRT3 plays a bifunctional role in cancer, either tumor-promoting or tumor-suppressing, depending on the cancer type and the status of intracellular signaling pathways (Xiong et al. 2016). Evidence suggests that SIRT3 may inhibit oxidative stress, thereby preventing cell death or, conversely, facilitating apoptosis (Torrens-Mas et al. 2017). It is believed that by examining the mechanistic variations across different cancer types, the carcinogenic and anticancer properties of SIRT3 can be revealed in greater detail. These findings may help to formulate innovative anticancer strategies targeted at SIRT3.

### Anticancer effect of ROS

As discussed above, elevated levels of ROS are essential for facilitating tumor initiation and progression. Paradoxically, excessive ROS formation can exert an antioncogenic effect (Perillo et al. 2020). The significant accumulation of ROS has the potential to inhibit the proliferation of tumor cells and induce apoptosis or necrosis, which can be attributed to the activation of various pathways, including endoplasmic reticulum stress, mitochondrial dysfunction, P53-mediated apoptosis, ferroptosis, and other mechanisms.

#### ROS-induced ferroptosis

**Fig. 6:**
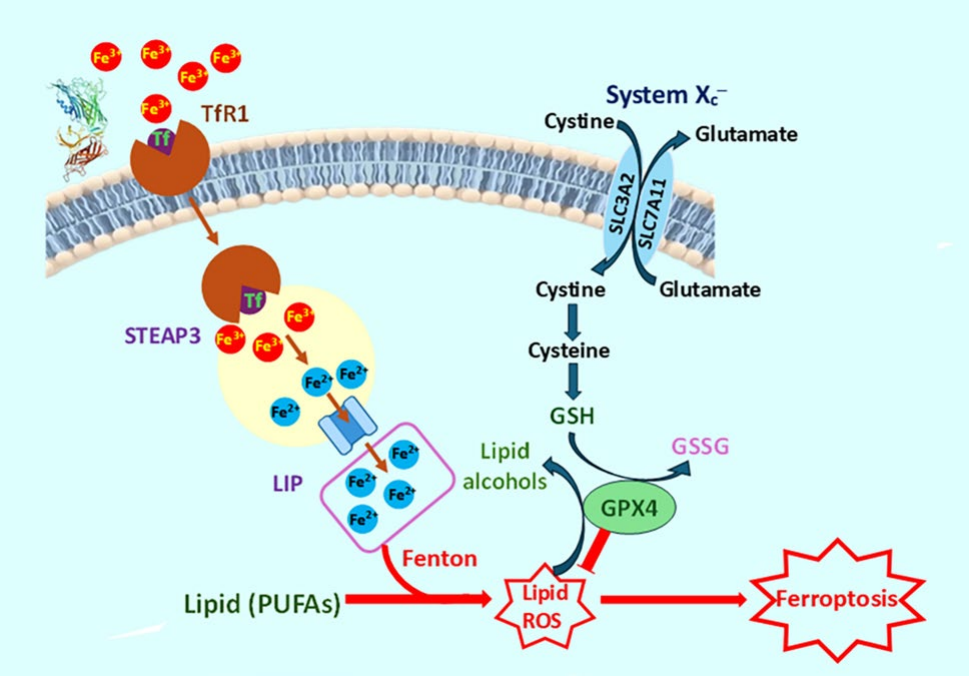
Simplified ferroptotic cascade. Transferrin receptor 1 (TfR1) is a type II transmembrane glycoprotein that interacts with transferrin (Tf) to facilitate essential cellular iron uptake in the ferric form, Fe^3+^. Fe^3+^ is liberated and subsequently reduced to Fe^2+^ by the metalloreductase STEAP3, which is capable of converting iron from an insoluble Fe^3+^ to a soluble ferrous Fe^2+^ form. Following reduction, Fe^2+^ is transported into the cytoplasm by divalent metal transporter 1 (DMT1), forming a labile iron pool (LIP). LIP can catalyze the formation of hydroxyl radicals via the Fenton reaction using hydrogen peroxide as a substrate. Hydroxyl radicals and other ROS are involved in the oxidation of polyunsaturated fatty acids (PUFAs), which form lipid peroxides. If lipid peroxides are not promptly eliminated within the cell, the continuous accumulation of lipid alkoxy (RO^•^) radicals ultimately results in ferroptosis. Ferroptosis can be inhibited by the glutathione peroxidase GPx4. This process requires glutathione (GSH) as a substrate provided by the cystine–glutamate transporter (System X_c_.^—^)

Ferroptosis represents a distinct type of iron-dependent regulated cell death that is nonapoptotic in nature and marked by the harmful buildup of membrane-localized lipids (Fig. 6) (Dixon And Olzmann 2024; Dixon et al. 2012). Numerous studies have shown that ferroptosis is crucial for eliminating tumor cells and suppressing tumor proliferation. Tumor cells have disturbed iron homeostasis, which is compensated for by the increased absorption of iron necessary for iron metabolism and proliferation, as well as for triggering ferroptosis. To cope with increased levels of oxidative stress, cancer cells exhibit increased synthesis of antioxidant enzymes responsible for lipid synthesis, which serve as substrates for the lipid peroxidation process. Glutathione peroxidase 4 (GPx4) is an enzyme that plays a significant role in inhibiting ferroptosis-mediated cell death, as it uniquely facilitates the conversion of lipid hydroperoxides into nontoxic lipid alcohols, thus preventing the lipid peroxidation process. This type of GPx4 mechanism of action is prevalent across various therapy-resistant states in cancer cells (Viswanathan et al. 2017; Fan et al. 2022). A variety of pharmacological agents, such as erastin and artesunate, which are capable of inactivating GPx4 via the inhibitory mechanism of the synthesis of reduced glutathione (GSH), can promote ferroptosis (Zhou et al. 2024).

#### ROS-mediated inhibition of the proliferative signaling pathway

The signaling pathways involving epidermal growth factor (EGF) and its receptor (EGFR) are essential for tumor development (Huang et al. 2021b). ROS can suppress the growth of cancer cells by interfering with these signaling pathways. When the concentration of ROS exceeds the capacity of cancer cells for self-regulation, it leads to a direct reduction in the expression of both EGF and EGFR, significantly inhibiting the phosphorylation of EGFR. Consequently, this disruption affects downstream signaling molecules associated with cell proliferation, including ERK and the PI3K/Akt pathway (Thamilselvan et al. 2017; Pei et al. 2019).

The prooxidant behavior of vitamin C is known to enhance ROS formation while simultaneously reducing the phosphorylation of ERK by lowering the release of epidermal growth factor (EGF) and the phosphorylation of EGF receptors. This mechanism leads to a decrease in the proliferation of thyroid cancer cells (Su et al. 2019). Similarly, koumine, an alkaloid isolated from Gelsemium elegans that possesses antitumor activity, has been shown to reduce the proliferation of hepatocellular carcinoma cells (Yuan et al. 2019). Furthermore, the application of H_2_O_2_ can hinder the phosphorylation of ERK1/2 in breast cancer cells in a dose-dependent manner, resulting in a reduction in cancer cell proliferation (Li et al. 2019).

Copper chaperone for superoxide dismutase (CCS) has been implicated as a potential tumor promoter in various cancers. CCS is significantly overexpressed in breast cancer, facilitating the proliferation and migration of these cells. The knockdown of CCS effectively decreases the phosphorylation levels of ERK1/2 and increases ROS production, ultimately resulting in the suppression of the proliferation and migration of breast cancer cells (Li et al. 2019).

Carmustine is a chemical drug that, together with selenite, significantly reduces the proliferation and survival of androgen-independent prostate cancer cells by disrupting EGFR signaling (Thamilselvan et al. 2017). Carmustine is an alkylating agent and is known under the brand name BiCNU (Ewend et al. 2007). In addition, the combination of these two agents, carmustine and selenite, triggered apoptosis in prostate cancer cells. The inhibitory effect of these agents on epidermal growth factor (EGF)-induced activation of epidermal growth factor receptor (EGFR), which is associated with downstream signaling, such as Akt, NF-κB, and ERK1/2, has been attributed primarily to increased ROS production.

Oenothein B is a macrocyclic polyphenol that has been shown to inhibit the proliferation of A549 lung cancer cells by triggering apoptosis and arresting cells at the G1 stage (Pei et al. 2019). A detailed study revealed that polyphenols not only significantly increased intracellular ROS levels but also induced the upregulation of intracellular apoptotic stimulants, such as caspase-3, an important protease that plays a key role in apoptosis; poly (ADP‒ribose) polymerase (PARP), a family of proteins involved in various cellular processes, including apoptosis; and Bax and Bak, members of the Bcl-2 family and core regulators of the intrinsic pathway of apoptosis. In summary, the polyphenol oenothein B prevented cell proliferation via the ROS-mediated PI3K/Akt/NF-κB signaling pathway.

#### ROS-induced cell death

Increased ROS levels can trigger cell cycle arrest, cellular senescence, and the death of cancer cells (Ichijo et al. 1997; Moon et al. 2010; Gao et al. 2023). ROS-induced cell death involves the activation of the apoptosis signal-regulating kinase ASK1/JNK and ASK1/p38 signaling cascades (Ichijo et al. 1997). In its inactive state, ASK1 binds to reduced thioredoxin (TRX), which can be oxidized by H_2_O_2_, resulting in the dissociation and subsequent activation of ASK1. This initiates the downregulation of antiapoptotic proteins via the downstream MAP-kinase kinase MKK4/MKK7/JNK and MKK3/MKK6/p38 pathways (Saitoh et al. 1998; Tobiume et al. 2001). Various tumors contain inactivating mutations in the JNK and p38 pathways, indicating that interference within these pathways may promote the death of tumor cells (Han et al. 2007). The ROS-induced activation of the p38 and JNK pathways can trigger cell cycle arrest in cancer cells (Wagner et al. 2009; Nakamura et al. 2014).

ROS-mediated endoplasmic reticulum (ER) stress pathways can induce cancer cell apoptosis. The ER is a major store of Ca^2+^, and increased ROS levels can trigger its release through the ER lumen. The release of Ca^2+^ from the ER affects mitochondrial metabolism and adjusts the threshold for apoptosis in response to chronic stress. Persistent ROS-induced ER stress activates inositol-requiring enzyme (IRE), a key regulator of ER stress that controls apoptosis in cancer cells (Redza-Dutordoir et al. 2016).

**Fig. 7:**
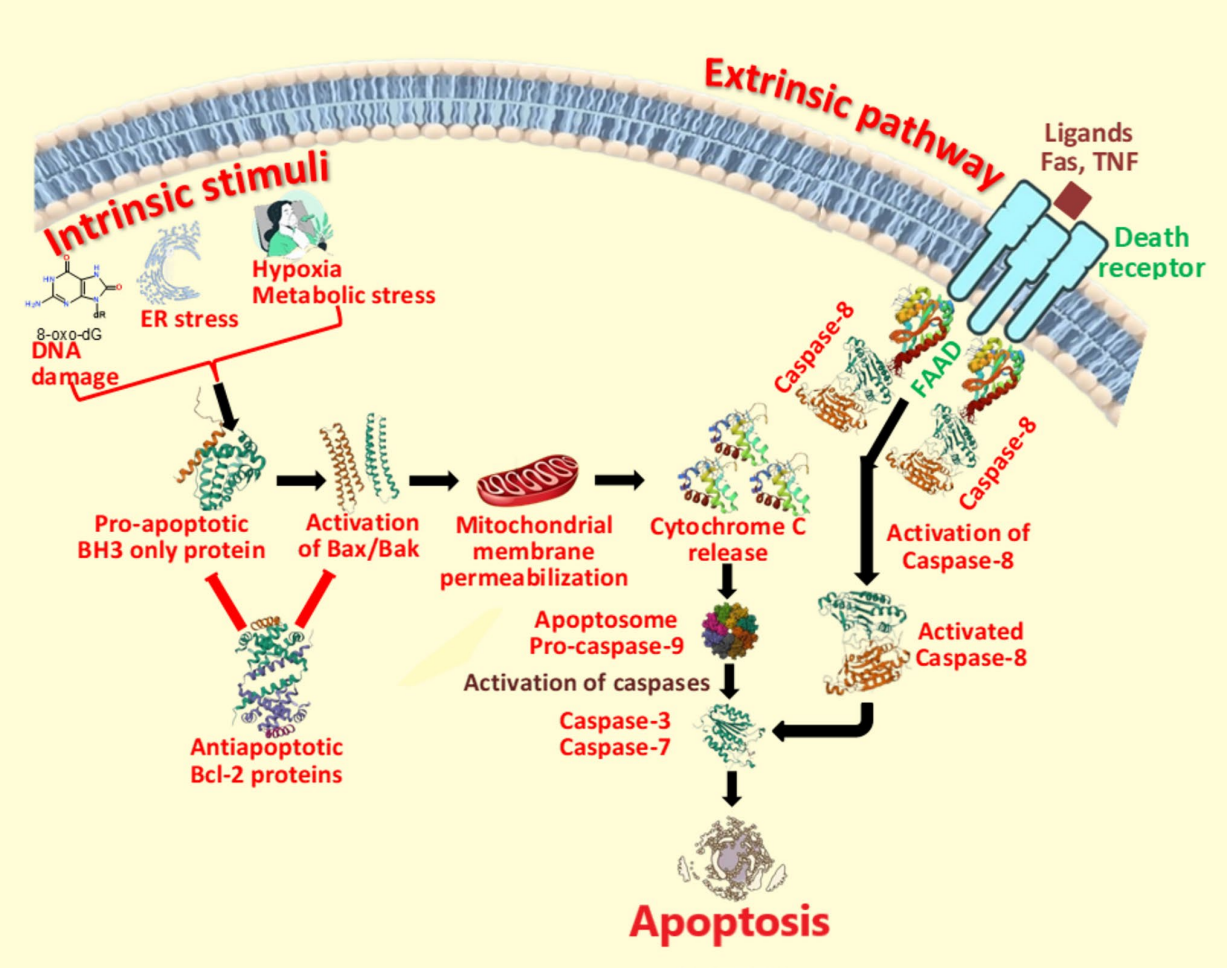
Molecular mechanisms of the intrinsic and extrinsic pathways of apoptosis. The intrinsic apoptotic pathway is initiated by internal stressors, such as DNA damage, endoplasmic reticulum (ER) stress, metabolic stress, or hypoxia. The proapoptotic BH3-only family members trigger the activation of proapoptotic Bax or Bak, resulting in the permeabilization of the mitochondrial outer membrane (MOMP) and cytochrome c release. This process facilitates the formation of the apoptosome, a protein complex involved in the intrinsic apoptotic pathway, which activates the initiator caspase-9 and consequently caspase-3 and caspase-7, ultimately leading to cell death. Proapoptotic BH3-only proteins can be inhibited via interactions with the antiapoptotic Bcl-2 family of proteins. In the extrinsic apoptotic pathway, ligands interact with death receptors, including Fas, a 40-kDa member of the tumor necrosis factor (TNF) family, and other apoptosis-inducing ligands. This interaction leads to the recruitment of Fas-associated death domain protein (FADD) and the subsequent activation of caspase 8. Activated caspase 8 then directly triggers the activation of caspases 3 and 7, which converge with the intrinsic pathway to induce cell death

The antitumor effect can be exerted via ROS-regulated mitochondrial apoptotic pathways (Fleury et al. 2002). Enhanced ROS formation is responsible for the permeabilization of the membrane, which in turn triggers the translocation of proapoptotic proteins/factors, including cytochrome c (Cyt C), from the mitochondria into the cytosol, representing the critical event of the mitochondrial apoptotic process. Approximately 20 members of the Bcl-2 family of proteins are classified as either proapoptotic or antiapoptotic. The integral components of the Bcl-2 family are Bax and Bak, which serve as essential regulators of the intrinsic apoptotic pathway. In response to apoptotic signals, Bax and Bak proteins become activated and form oligomers at the mitochondrial outer membrane, facilitating their permeabilization (Fig. 7) (An et al. 2024).

Other proapoptotic factors include apoptosis-inducing factor mitochondria-associated 1 (AIFM1) and diablo IAP-binding mitochondrial protein (DIABLO/SMAC) (Peña-Blanco et al. 2018). The release of cytochrome c into the cytoplasm triggers the activation of initiator caspase 9 (CASP9), followed by the sequential activation of caspase 3 (CASP3) and caspase-7 (CASP7), known as executioner caspases (Fig. 7).

α-Hederin is a pentacyclic triterpene saponin with anti-inflammatory, antioxidant, antiviral, and antitumor properties against various human cancer cell lines (Adamska et al. 2019). α-Hederin has been shown to induce excessive ROS formation, resulting in increased cytochrome c in the cytosol; decreased levels of Bcl-2; and increased levels of Bax, caspase-3 and caspase-9, confirming that α-hederin induces apoptosis via the mitochondrial pathway (Wang et al. 2020).

A variety of metal-based drugs show great potential as antitumor agents. Among these, metal–thiourea complexes have garnered significant interest because of their unusual biological properties. Au(I)–thiourea complexes contain Au atoms coordinated by two sulfur atoms and exhibit remarkable cytotoxicity against various tumor cell lines, as demonstrated by significantly increased ROS levels and disrupted mitochondrial membrane potential (MMP), which in turn leads to the release of cytochrome c and subsequent activation of caspases 9, 7, and 3 (Yu et al. 2020). Mechanistic studies revealed that Au (I)–thiourea-based complexes downregulated Bcl-2 and upregulated Bax, suggesting that apoptosis is induced through an ROS-induced mitochondria-mediated pathway.

#### P53-dependent apoptosis

*TP53* is a gene encoding the p53 protein, consists of six major domains, functions as a tumor suppressor, and is often described as the"Guardian of the Genome” (Lane 1992; Hernández Borrero And El-Deiry 2021). The p53 protein not only plays a critical role in tumor suppression but also significantly influences the response of both malignant and nontransformed cells to various chemotherapies, especially those that induce DNA damage (Aubrey et al. 2018). p53 performs multiple functions in response to DNA damage. These functions include facilitating DNA repair, inducing cell cycle arrest, promoting senescence, and triggering apoptosis, all of which serve to prevent the transmission of mutations.

Exposure of cells to elevated levels of H_2_O_2_ or other ROS is manifested by both the stabilization of p53 and the activation of the cellular DNA damage response. Given that DNA damage is a well-established precursor to p53 activation, it has been inferred that DNA damage resulting from ROS is the primary factor leading to the downstream signaling pathways that stabilize p53. However, some ROS and H_2_O_2,_ in particular, function not only as damaging species but also as signaling molecules, triggering pathways, such as the JNK/p38MAPK pathway, which can activate p53 independently of the DNA damage response. Therefore, distinguishing between the DNA damage response, redox signaling activation, or even their combination in triggering p53 activation is difficult (Shi And Dansen 2020a, 2020b).

Piperlongumine (PL), an alkaloid derived from black pepper (Piper longum), has been shown to induce apoptosis in tumor cells (Basak et al. 2016). Two human colon cancer cell lines, HT29 and SW620, both containing mutated p53, were treated with piperlongumine. Treatment of both cancer cell lines significantly increased ROS production, the level of protein glutathionylation, the expression of Nrf-2, and the expression of p53 target genes, including BAX. Given that p53 is a protein sensitive to redox changes, it has been hypothesized that the prooxidant environment induced by piperlongumine promotes functional recovery of mutant p53 via protein glutathionylation, ultimately resulting in the apoptosis of human cancer cell lines.

R-goniothalamin is a plant secondary metabolite with anticancer and apoptotic properties. The significant cytotoxic effect of this plant metabolite on breast cancer cells was attributed to the induction of oxidative stress and the reactivation of mutant p53 (Punganuru et al. 2018). In studies conducted in nude mice, a significant delay in tumor growth was observed with the administration of R-goniothalamin alone or in combination with cisplatin.

In addition to organic-based agents, redox-metal-bearing complexes, owing to their redox properties and relatively low toxicity, have become attractive targets in the design of potential anticancer drugs (Valko et al. 2016). Treatment of hepatocellular carcinoma cells with a Cu(II) complex containing a triphenyl-phosphine (TPP) ligand, [Cu(ttpy-tpp)Br_2_]Br, induced significant ROS generation, which resulted in disrupted mitochondrial membrane potential, the aggregation of Bax in mitochondria, and the release of cytochrome c (Shao et al. 2019). These findings indicate that the Cu complex induced apoptosis in human hepatocellular carcinoma cells via the mitochondrial pathway. Underlying mechanisms revealed that the ROS-induced translocation of p53 into the mitochondria represents a key step in the mechanism of apoptosis. The survey of apoptotic pathways is outlined in Fig. 7 (Ahmad Bhat et al. 2025).

#### ROS-mediated inhibition of metastasis

For unknown reasons, solid tumors entering the bloodstream have a limited capacity to establish distant metastases. Human melanoma cells are normally characterized by unusually high metastatic potential. In contrast to those in subcutaneous melanoma, the cells of this type in the blood and visceral (soft) organs experience increased levels of ROS-induced oxidative stress, which is reflected in their reduced ability to metastasize (Piskounova et al. 2015). The administration of antioxidants that suppress oxidative stress increased the distant metastatic potential of melanoma cells. Oxidative stress thus plays a critical role in limiting the metastatic capacity of melanoma cells.

## Oxidative stress changes in different stages of carcinogenesis

During the initiation stage of cancer, which is characterized by increasing levels of oxidative stress, genetic alterations facilitate cellular survival (adaptation to oxidative stress) via the activation of antioxidant transcription factors and/or the increase in NADPH production, which is required for fueling the antioxidant machinery (Iqbal et al. 2024).

**Fig. 8:**
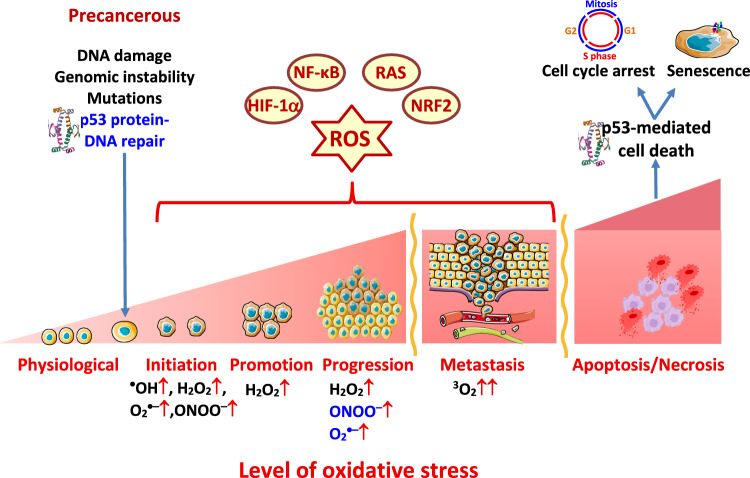
Schematic view of the cancer development progressing through a multistep process from tumor initiation to metastasis**.** ROS marked in blue (ONOO^−^, O_2_^•−^), have an inhibitory effect on cancer progression. The increased levels of ROS and/or antioxidants result in a disturbed redox balance that promotes the activation of intracellular oncogenic signaling pathways and sustains the functionality of cellular components within cancer cells. Abbreviations: transcription factor hypoxia-inducible factor 1 alpha (HIF-1α), nuclear factor erythroid 2-related factor 2 (NRF2), nuclear factor kappa B (NF-κB), rat sarcoma (RAS)

ROS/RNS not only initiate this process but also facilitate the proliferation of already initiated cells during the promotion and progression phases of tumor development (see Fig. 8). The initiation phase of carcinogenesis is characterized by a sustained attack of genotoxic species, such as O_2_^•—^, ^•^OH, ONOO^—^ and H_2_O_2,_ resulting in the formation of genetic changes necessary for cell growth. The promotion stage of cancer is characterized by H_2_O_2_-mediated cell proliferation of preneoplastic nodules that have developed the capacity for independent growth (Hayes et al. 2020). The characteristic feature of the progression phase is the local invasion of autonomously growing cancer cells into adjacent healthy tissue. In addition, the interaction of cancer cells with stromal and immune cells is typical for this cancer stage. During the progression stage, H_2_O_2_ is involved in the epithelial-to-mesenchymal transition (EMT) required for metastatic spread and the protumorigenic activity of tumor-associated macrophages, which participate in the formation of the tumor microenvironment. However, O_2_^•—^ and ONOO^—^ are most likely engaged by immune cells to kill tumor cells. Metastasis is characterized by the spread of cancer cells into the bloodstream or lymphatic system and survival in the circulatory system to colonize distant organs. During this stage, the role of antioxidants is unclear, and high levels of ROS may trigger apoptosis in various types of cancer cells.

Given that tumorigenesis requires constant ROS flux, understanding how cells can cope with high levels of protumorigenic ROS and maintain a shifted redox balance without triggering senescence or apoptosis is key. In this context, many upregulated antioxidant genes can mediate adaptation to oncogene-triggered oxidative stress (see below).

### DNA damage as a source of genomic instability

Increased mutation rates characterize genomic instability and can be considered a paradoxical phenomenon. While mutations serve as catalysts for genetic diversity and are instrumental in the process of natural selection, they also pose significant risks, such as the incidence of chronic diseases, including cancer (Tubbs And Nussenzweig 2017). The emergence of mutations can be attributed to the failure of DNA repair mechanisms.

It is widely recognized that prolonged exposure to elevated levels of ROS can lead to oxidative DNA damage associated with a COSMIC (catalog of somatic mutations in cancer) mutation signature (Rose et al. 2020). The most compelling evidence suggesting that ROS may increase cancer risk has been confirmed by the knockout of O_2_^•^–scavenging enzymes, such as cytoplasmic Cu, Zn-SOD null mice, or mitochondrial Mn-SOD null mice, resulting in significant oxidative damage and a propensity to develop cancer spontaneously (Gill et al. 2016).

Oxidative stress is a key driver of mutagenesis and tumorigenesis through oxidative DNA damage and a significant phenomenon in the conversion of normal cells into cancerous phenotypes, primarily by undermining genomic integrity (Bartek 2011). Cellular targets of ROS-induced oxidative stress involve cellular macromolecules, such as DNA, RNA, proteins, and membrane lipids. ROS-induced DNA mutations, strand breaks, and chromosomal abnormalities through their interaction with nitrogenous bases and the sugar‒phosphate backbone are fundamental contributors to the onset of cancer. Genetic modifications disrupt the normal regulatory processes of the cell cycle, apoptosis, and DNA repair mechanisms.

The key mutation involves the oxidative modification of guanine to form 8-oxoguanine (8-oxo-G) in DNA, termed 8-oxo-7-hydro2’-deoxyguanosine (8-oxo-dG) (see Fig. 3) (Hahm et al. 2022). The initial identification of 8-oxoguanine in DNA occurred during the analysis of carcinogenic compounds associated with oxidative stress; consequently, 8-oxoguanine has become a widely recognized biomarker for assessing intracellular oxidative stress levels [Dizdaroglu et al. 2002; Jaruga et al. 2001]. 8-Oxoguanine can be generated directly at the levels of DNA (8-oxo-dG) and RNA (o^8^G) or at the level of free nucleotides (8-oxo-dGTP or o^8^GTP), which may subsequently be incorporated during DNA replication or RNA transcription.

This adduct is significantly elevated in various malignant tumor tissues compared with surrounding healthy tissues (Chiorcea-Paquim 2022). The accumulation of DNA damage disrupts the interpretation and transmission of genetic information, resulting in permanent alterations to the genetic material, which is a crucial step in the processes of mutagenesis and tumorigenesis.

ROS can also induce depurination and depyrimidination and cause both single-strand and double-strand breaks in DNA. If double-strand breaks are not properly repaired or are misrepaired, they can result in chromosomal instability, cell death, and the incidence of cancer (Chiorcea-Paquim 2022). Nevertheless, the association between low levels of ROS and the generation of double-strand breaks remains a topic of debate, partly owing to methodological challenges, such as those encountered with the neutral Comet assay and the use of nonspecific biomarkers for double-strand breaks, such as gamma-H2AX, an early cellular response to the induction of DNA double-strand breaks. Double-strand breaks may also occur as repair intermediates during the resolution of oxidatively induced clustered DNA lesions (Sage and Harrison 2011), consisting of closely located lesions, typically within 20 base pairs, which include abasic (AP) sites, oxypyrimidines, oxypurines, and single-strand breaks (Kryston et al. 2011).

Genomic mutations can arise during the S phase of the cell cycle. A critical step is DNA replication, during which DNA polymerases, which are responsible for DNA synthesis, may introduce small but fatal errors into the whole process, such as the incorporation of incorrect nucleotides into DNA. In addition, DNA polymerases are susceptible to a phenomenon termed “stuttering”, which is characterized by extensive nucleotide repeats.

The concept that hydroxyl radical (^•^OH)-mediated DNA damage and cancer incidence increase the probability of tumor development is supported by hemochromatosis, characterized by iron overload, which catalyzes the formation of ^•^OH via the Fenton reaction (Torti et al. 2018). In the context of chronic inflammation, increased concentrations of peroxynitrite (ONOO^—^) can interact with carbon dioxide (CO_2_), resulting in the formation of nitrosoperoxycarbonate (ONOOCO_2_^−^) (Jomova et al. 2024). The decomposition of nitrosoperoxycarbonate into CO_3_^•^—and nitrogen dioxide ^•^NO_2_ can initiate the selective oxidation and nitration of guanine, finally forming guanine‒thymidine crosslinks (Shafirovich And Geacintov 2017).

A reduced antioxidant capacity, which is associated primarily with glutathione, also causes DNA damage and represents an increased risk of tumorigenesis. Glutamate–cysteine ligase (GCL) is the rate-limiting enzyme for glutathione synthesis, and knockout of the mouse Gcl gene is associated with embryonic lethality and increased risk of carcinogenesis (Yang et al. 2002).

### Oxidative stress and counteracting mechanisms in the initial stages of carcinogenesis

The initial phase of tumorigenesis is characterized by activated oncogenes and increased metabolic demands, leading to elevated intracellular ROS levels in early neoplastic lesions. ROS-mediated damage has been associated with O_2_^•—^ production by mitochondria and NADPH oxidase (NOX) and H_2_O_2_ production by 5-lipoxygenase and the endoplasmic reticulum (Arnandis et al. 2018; Raimondi et al. 2020).

Oncogene-mediated ROS production has been associated with (i) Ras-activated signaling altering the metabolism of the mitochondrial potential and increasing the activity of NADPH oxidases 2 and 4 (NOX2 and NOX4), (ii) antiapoptotic B-cell lymphoma 2 (Bcl-2) altering the mitochondrial potential, (iii) signal transducer and activator of transcription 3 (STAT3) altering mitochondrial metabolism and activating NADPH 4 (NOX4), and (iv) the multipurpose oncogenic transcription factor MYC (a cancer of the myelocytes, myelocytoma), which downregulates mitochondrial mass and biogenesis (Hayes et al. 2020; Ingelmann et al. 2019; Marcar et al. 2019).

Cancer cells can also be induced to generate ROS through the activity of tumor necrosis factor-alpha (TNF-α), which is released by immune cells. Alternatively, cancer cells may be exposed to ROS produced by immune cells that are attracted to the tumor microenvironment. Increased ROS levels in cancer cells have the capacity to suppress the activity of mitogen-activated protein kinase (MAPK), protein tyrosine phosphatases (PTPs), phosphatases, and tensin homolog (PTEN), which in turn may promote both cell proliferation and survival (Moloney And Cotter 2018).

To capitalize on the proliferative advantages conferred by elevated ROS levels while reducing the probability of senescence or apoptosis, tumor cells increase the expression of antioxidant transcription factors and/or modify their metabolic processes. The ability of cancer cells to prevent apoptosis via increased ROS formation requires increased antioxidant defense, such as increased transcription of genes encoding thioredoxin (TXN)- and glutathione (GSH)-dependent enzymes, as well as other detoxification and antioxidant enzymes (Fig. 1) (Harris et al. 2015). This redox shift involves suppressed gene repression by KEAP1, manifested by induced NRF2 target genes. In addition, a shift in the redox state increases the mRNA expression of HIF target genes, including glucose transporter type 1 (GLUT1), monocarboxylate transporter 4 (MCT4), and hexokinase 2 (HK2). In addition, NF-κB activity may be involved in adjusting the redox state via increased antioxidant protein expression (Koch et al. 2020). While the induction of apoptosis in the early stages of tumorigenesis requires simultaneous inhibition of both glutathione- and thioredoxin-based antioxidant systems, in the case of premalignant cells, the inhibition of only glutathione synthesis by various agents, including auranofin, sulfasalazine, buthionine sulfoximine, and alpha-methyl buthionine sulfoximine, is sufficient (Hayes et al. 2020).

Other mechanisms are involved in increasing NADPH levels and facilitating the de novo synthesis of glutathione (GSH) and antioxidant enzymes through various mechanisms. An important ROS regulator in cancer cells is the transcription factor nuclear factor erythroid 2-related factor 2 (NRF2), whose activity is modulated by Kelch-like ECH-associated protein 1 (KEAP1), a ubiquitin ligase that mediates the degradation of NRF2 (see also above). Increased ROS levels inhibit the KEAP1-mediated degradation of NRF2, which triggers a protective antioxidant defense. NRF2 promotes the expression of many antioxidant enzymes, including those involved in glutathione biosynthesis carried out by glutamate-cysteine ligase (GCL), which is composed of catalytic (GCLC) and modifier (GCLM) subunits (Franklin et al. 2009), and the regeneration of cytosolic NADPH from NADP via three routes, including malic enzyme 1 (ME1), isocitrate dehydrogenase 1 (IDH1), and the oxidative pentose phosphatase pathway (oxPPP) (Chen et al. 2019).

As discussed above, NRF2 is a clear example of the dual role of antioxidants in cancer. In the early stages of malignancy, NRF2 inhibits cancer cells in liver and urinary bladder animal models (Ramos-Gomez et al. 2001); conversely, during the later stages of tumor development, characterized by high ROS levels, NRF2 appears to be a protective barrier against ROS-induced cell death in several other model systems, including lung and pancreatic cancers (DeNicola et al. 2011; Romero et al. 2017). Enhanced NRF2 activation and its protective effects against ROS have also been observed in multiple human cancer types, often due to mutations in the KEAP1 gene (Romero et al. 2017). Notably, oncogenes, such as KRAS, BRAF, and MYC, which are associated with elevated reactive oxygen species (ROS) production, also stimulate compensatory antioxidant mechanisms (Son et al. 2013). These mechanisms may involve enhanced cystine transport and upregulation of NRF2 expression.

An important ROS regulator in cancer, the tumor suppressor protein p53, has both antioxidant and prooxidant properties (Bensaad et al. 2006; Kang et al. 2013). Prooxidant behavior manifested by p53-mediated ROS formation can trigger cell death via apoptosis or ferroptosis (Jiang et al. 2015), whereas antioxidant behavior is believed to act as a tumor suppressor by mitigating the accumulation of cellular damage (Huo et al. 2017). Paradoxically, the ROS-limiting capabilities of p53 have also been implicated in facilitating tumor growth by suppressing excessive oxidative stress, which could cause cancer cell death (Humpton And Vousden 2016). Notably, certain point mutations of p53 that are frequently observed in various cancers appear to maintain the capacity to protect cells from ROS-mediated destruction (Humpton et al. 2018).

As the hypoxic microenvironment is a prevalent and notable characteristic of most solid tumors, hypoxia significantly influences the biological behavior and malignant characteristics of cancer cells (Chen et al. 2023). Adaptation to hypoxia, metabolic rates, oncogenic mutations, and protumor signaling activation can all contribute to tumorigenesis. Cancer cells respond to hypoxic conditions by modifying their metabolic processes, primarily through the activation of key genes such as hypoxia-inducible factors (HIFs). Hypoxia-mediated pathways can significantly influence the malignant behavior of cancer cells, with most downstream targets being dependent on HIFs. Hypoxia can alter the properties of mesenchymal stromal cells within the tumor microenvironment. In addition to intrinsic components, the tumor microenvironment (TME) has external elements, such as the microbiota, which are important factors affecting the development of various cancers. The microbiota within the TME can induce chronic hypoxia, activate HIF-1α, and ultimately induce the expression of regulatory genes associated with HIF-1α.

### Loss of tumor suppression manifested as downregulated expression of antioxidant enzymes

Increased levels of damaging ROS in cancer cells can inactivate the tumor suppressor protein p53, which in turn results in decreased expression of key antioxidant enzymes, such as superoxide dismutase (SOD), glutathione peroxidase (GPx), and sestrin 1 (SESN1), known as the p53-regulated protein PA26.

Studies have shown that mice lacking peroxiredoxin-1 (PRDX1), Cu, Zn-SOD (SOD1) or Mn-SOD (SOD2) present increased incidences of various malignancies (Neumann et al. 2003; Busuttil et al. 2005; Van Remmen et al. 2003). Conversely, upregulated Mn-SOD (SOD2) expression inhibits tumor progression in a mouse model of T-cell lymphoma. Modifying antioxidant enzymes such as SOD2 acetylation has been shown to increase mitochondrial ROS, promoting hypoxic conditions and a prooxidant environment favorable for cancer (Gorrini et al. 2013).

In addition, the deletion of one or more glutathione peroxidase (Gpx) genes, specifically Gpx1–Gpx3, has been linked to increased vulnerability to cancer in mice (Chu et al. 2004). The observed sensitivity indicates that peroxidative stress is a significant factor in the pathology and inflammation of the ileum and colon, potentially contributing to the development of tumors.

Furthermore, the downregulation of Gpx3 is frequently observed in human cancers, which aligns with its proposed role as a tumor suppressor (Barrett et al. 2013). Moreover, the involvement of increased expression of glutathione S-transferases (GSTs) in cancer progression and drug resistance has also been documented.

## Dichotomy of ROS in metastasis

ROS are integral to multiple facets of tumor metastasis, including proliferation, invasion, angiogenesis, inflammation, and immune evasion. Research indicates that ROS play a dual role in cancer metastatic progression and are capable of either supporting or inhibiting this process (Chen et al. 2024). The role and impact of ROS in metastasis, determining whether they facilitate or hinder this process, depend upon the type of tumor, the type of ROS species, the site of ROS generation within the tumor, the level of ROS, the difference between the antioxidant and prooxidant response, and other factors (Aggarwal et al. 2019). For example, hydrogen peroxide generated by mitochondrial ROS can facilitate metastasis (Goh et al. 2011; Perillo et al. 2020), whereas lipid peroxides resulting from the oxidation of membrane lipids may compromise cell survival during the metastatic process (Ubellacker et al. 2020).

### Metastatic potential of ROS

Cancer cells do not operate in isolation during various metastatic phases; rather, they act in a concerted fashion with adjacent tissues, the extracellular matrix, immune cells, blood cells, and endothelial cells (Nishikawa 2008). This interaction induces modifications within the cancer cells themselves and the surrounding noncancerous cells, resulting in altered expression of numerous genes.

Growing tumors gradually infiltrate adjacent tissues and spread to remote organs. Compared with proliferation at the primary site, metastasis requires additional specificities, such as migration and invasion capacity (Fares et al. 2020). The development of metastasis requires that cancer cells detach from their original location, traverse the bloodstream, withstand pressure within blood vessels, adapt to the cellular environment of a secondary site, and evade lethal interactions with immune cells. According to Hanahan and Weinberg, the activation of invasion and metastasis represents the characteristics of cancer (Hanahan and Weinberg 2011). All stages of metastatic progression are characterized by dual ROS functioning, either facilitating or hindering individual metastatic stages.

ROS-induced activation of HIF-1α in a murine model of mammary cancer activated several genes required for efficient tumor growth and lung metastasis. The administration of an antioxidant food additive, butylated hydroxyanisole, mitigated the metastatic process (Chourasia et al. 2015).

During extracellular-matrix separation, receptor-interacting protein kinase 1 (RIPK1) triggers mitochondrial autophagy via a pathway reliant on the mitochondrial phosphatase PGAM5, which regulates mitochondrial dynamics to influence cellular senescence (Hawk et al. 2018). This process results in suppressed levels of NADPH, which in turn leads to an increase in ROS concentrations.

Furthermore, ROS can influence the NF-κB signaling pathway. In chronic inflammatory settings, ROS predominantly arise from activated immune cells, whereas inflammatory mediators can further increase ROS production within these cells. Activated oncogenes are associated with increased ROS levels, which may impede DNA replication. Thus, ROS can facilitate tumor metastasis either through direct damage or indirect modulation of cellular proliferation or survival signaling pathways, including the NF-κB pathway.

### Dissemination and invasion

The invasion‒metastasis cascade is triggered by chromosomal instability induced during mitosis. The release of genomic DNA into the cytosol activates cyclic GMP–AMP synthase, which is accompanied by the activation of nuclear factor kappa-light-chain enhancer of activated B cells (NF-κB) signaling (Bakhoum et al. 2018). Successful cancer cell seeding requires the coordinated movement of a cluster of tumor cells, highlighting the importance of epithelial‒mesenchymal transition (EMT) in this process.

#### Epithelial‒mesenchymal transition (EMT)

The epithelial‒mesenchymal transition (EMT) plays a pivotal role in facilitating tumor invasion and metastasis. Epithelial cells are typically nonmotile and adhere to one another and the surrounding extracellular matrix (ECM) (Fouad And Aanei 2017). The epithelial‒mesenchymal transition is governed by biochemical changes that allow specific epithelial cells to adopt a mesenchymal phenotype, acquire invasive capabilities, disseminate throughout the body, and resist stress (Ye and Weinberg 2015). The epithelial‒mesenchymal transition confers epithelial cell plasticity, which is essential for cancer cell progression and metastatic capacity, although only some cells of the primary tumor show metastatic potential.

Elevated ROS levels, triggered by various cancer-associated signals, can stimulate EMT. In a model of pancreatic ductal adenocarcinoma (PDAC), increased ROS levels significantly promoted EMT and enhanced migration, invasion, and metastatic potential (Cheung et al. 2020).

Cancer-associated fibroblasts are the key elements of epithelial-to-mesenchymal signatures in the tumor microenvironment, and their additional ROS support facilitates epithelial‒mesenchymal transition, thereby increasing the invasiveness of tumor cells and promoting metastasis (Szabo et al. 2023).

Increased concentrations of ROS can trigger the activation of nuclear factor-κB (NF-κB), leading to the upregulation of the Snail superfamily of zinc-finger transcription factors. This process results in the downregulation of epithelial cadherin (E-cadherin or cadherin-1) while simultaneously enhancing the expression of neural cadherin (N-cadherin or cadherin-2) and vimentin, an intermediate filament protein (Radisky et al. 2005). The loss of function of E-cadherin is associated with cancer progression by enhancing proliferation, invasion, and metastasis, and the shift toward increased expression of N-cadherin contributes to the disruption of intercellular junctions and initiates the process of epithelial‒mesenchymal transition (Jiang et al. 2017). In addition to the activation of NF-κB, ROS can activate hypoxia-inducible factor 1α (HIF-1α), facilitating the transcription of Snail and resulting in the downregulation of E-cadherin.

It has been reported that exposure to sulfate aerosols induces the invasion and migration of lung epithelial cells and that ammonium sulfate (NH_4_)_2_SO_4_ promotes lung tumor metastasis in vivo (Yun et al. 2017). This was associated with increased levels of ROS, further facilitating the transcription of Snail induced by HIF-1α, resulting in the downregulation of E-cadherin.

Mitochondrial calcium uniporter regulator 1 (MCUR1) is often found to be significantly upregulated in hepatocellular carcinoma, supporting the survival of cancer cells. It has been reported that highly expressed MCUR1 promotes in vivo metastasis by inducing epithelial‒mesenchymal transition via Snail (Jin et al. 2019). Closer inspection of the mechanism revealed that MCUR1-mediated mitochondrial Ca^2+^ signaling facilitated epithelial‒mesenchymal transition by activating the ROS/Nrf2/Notch1 signaling pathway. Decreased ROS formation markedly reduced the epithelial‒mesenchymal transition induced by MCUR1 in hepatocellular carcinoma cells.

The protease La (known as the Lon protein) is a homo-oligomeric ATP-dependent protease that is responsible for peptide bond cleavage. Upregulation of this protein increases mitochondrial ROS, which in turn activates NF-κB and ROS-dependent p38 signaling, resulting in the promotion of the process of epithelial‒mesenchymal transition (Kuo et al. 2020). Mitochondrial Lon triggers the production of ROS-dependent inflammatory cytokines, which in turn stimulate angiogenesis (Chen et al. 2024a, 2024b).

Many investigations have demonstrated that increased membrane- and mitochondrial-derived ROS triggered by various cancer-related signals promote epithelial‒mesenchymal transition (Cheung et al. 2020).

The application of the antioxidant N-acetyl-l-cysteine (NAC) to cancer cells has been shown to facilitate a switch back to an epithelial phenotype (Li et al. 2020a, 2020b). In addition, epithelial‒mesenchymal transition has been linked to the emergence of cells exhibiting a more progenitor-like, self-renewing phenotype, referred to as cancer stem cells, which are believed to play crucial roles in several significant clinical features of cancer. Interestingly, owing to the increased antioxidant pool, certain cancer stem cells have suppressed ROS levels, increasing their resistance to ROS-mediated damage (Diehn et al. 2009).

#### Migration/invasion

Cell migration and invasion represent critical survival mechanisms employed by cancer cells to evade various stresses within the tumor microenvironment (TME) (Kuo et al. 2022). The enhancement of migratory capabilities, driven by epithelial‒mesenchymal transition (EMT) processes, is facilitated through ROS-mediated pathways involving MAPK/NF-κB, as induced by the mitochondrial Lon protease (Kuo et al. 2020).

One of the most prominent cytokines released by overexpressed cancer cells is Transforming growth factor-beta (TGF-β), which occurs in both cancer cells and the surrounding microenvironment (Kuo et al. 2020). TGF-β-mediated signaling and cell migration modulated by NF-κB have been associated with tumor-promoting properties in non-Hodgkin’s cutaneous T-cell lymphoma (CTCL) and other predominantly solid tumors (Chang et al. 2015). TGF-β1 is well-known not only to trigger mitochondrial ROS formation but also to activate genes related to EMT (Ishikawa et al. 2014).

Induced ROS production by overexpressed mitochondrial Lon protease enhances the expression of TGF-β via NF-κB signaling, which in turn affects the immune suppressive microenvironment necessary for TGF-β-mediated epithelial‒mesenchymal transition and inflammatory responses (Kuo et al. 2020; Lamouille et al. 2014). Thus, the mitochondrial Lon protease can activate downstream ROS-mediated signaling to increase tumorigenesis and metastasis.

Elevated levels of both membrane and mitochondrial ROS activate matrix metalloproteinases, a large family of Ca-dependent and Zn-containing endopeptidases (Nelson And Melendez  2004; Cheung And Vousden 2022). Matrix metalloproteinases interfere with all stages of cancer and can degrade the extracellular matrix, which allows cancer cells to infiltrate surrounding tissues (Mori et al. 2019). The increased activity of matrix metalloproteinases, accompanied by increased Mn-SOD, has been associated with increased migration and metastasis, confirming the importance of Mn-SOD in cancer invasion and metastasis (Hemachandra et al. 2015). This process was mitigated by the expressed H_2_O_2_-converting enzyme glutathione peroxidase (GPx), suggesting a role for H_2_O_2_ in breast cancer. The uptake of H_2_O_2_ from the extracellular environment through the water membrane transporter aquaporin 3 (AQP3) further facilitates the migration and invasion of breast cancer cells (Satooka et al. 2016). Increased levels of aquaporin 3 expression are typical of a poor prognosis in various cancers.

A molecular mechanism through which mutations in the Keap1‒Nrf2 pathway facilitate metastasis has been reported (Lignitto et al. 2019). Experiments revealed that activated Nrf2 prevents the Fbxo22-mediated degradation of Bach1 by inducing the expression of heme oxygenase (HO-1). Lung cancer cells with elevated Nrf2 levels simultaneously exhibit increased Bach1 levels, with the latter enhancing metastasis by activating the transcription of prometastatic genes.

A mouse model of lung cancer confirmed that low ROS levels facilitate the spread of tumors by stabilizing Bach1. The administration of antioxidants further increased the expression of Bach1 under conditions of suppressed oxidative stress, which is associated with migration and metastasis (Wiel et al. 2019). This finding confirms that increased levels of ROS increase the degree of invasiveness of cancer cells.

Research utilizing animal models of melanoma (Le Gal et al. 2015a, 2015b) and lung cancer (Sayin et al. 2014) confirmed that antioxidant support and/or genetic alterations can increase metastatic potential. These findings indicate that the administration of the strong antioxidant N-acetylcysteine (NAC) enhances lymph node metastases in an endogenous mouse model of malignant melanoma but does not influence the quantity or dimensions of primary tumors (Le Gal et al. 2015a, 2015b). The combined effect of NAC and the soluble vitamin E derivative Trolox significantly augmented the migratory and invasive capabilities of human malignant melanoma cells.

B-RAF and K-RAS are two key oncogenes in the RAS/RAF/MEK/MAP-kinase signaling pathway, and their double mutations and functional classes of BRAF mutations are found in non-small cell lung cancers (NSCLCs). N-acetylcysteine and vitamin E significantly enhance the development of B-RAF- and K-RAS-induced mouse models of lung cancer and decrease survival rates (Sayin et al. 2014). The antioxidants NAC and vitamin E promoted tumor cell proliferation by suppressing ROS, minimizing DNA damage, and suppressing p53 expression in both mouse and human lung tumor cells. The inactivation of p53 results in tumor growth that parallels the effects of antioxidants. Thus, antioxidants facilitate tumor growth by interfering with the ROS‒p53 regulatory pathway. Given that somatic mutations in p53 typically arise later in tumor development, antioxidants may increase the progression of early tumors or precancerous lesions in high-risk groups, including smokers.

#### Extracellular–matrix detachment

Alterations in glucose metabolism triggered by the detachment of the extracellular matrix can lead to a bioenergetic deficit, which may activate pathways associated with cell death. Concurrently, cells that are detached from the extracellular matrix are exposed to elevated levels of ROS, which can also promote cell death.

Cancer cells detached from the extracellular matrix employ various molecular strategies to increase glucose metabolism and ensure their survival (Mason et al. 2017). One notable mechanism involves the upregulation and/or activation of receptor tyrosine kinases (RTKs), leading to the phosphorylation of glucose transporters and their subsequent relocation to the plasma membrane (Schafer et al. 2009). Furthermore, cancer cells with oncogenic mutations in Ras (rat sarcoma virus) exploit downstream signaling pathways via PI(3)K to support glucose uptake and survival during extracellular-matrix detachment.

Detached cancer cells encounter increased concentrations of ROS, which, if not regulated, may lead to apoptosis (Davison et al. 2013). This can be attributed, in part, to the inhibition of fatty acid oxidation mediated by ROS, resulting in a bioenergetic deficit in the detached cells. Nevertheless, re-establishing glucose uptake through oncogene activation during extracellular-matrix detachment can facilitate glucose flux through the pentose phosphate pathway (PPP). The resulting upregulation of NADPH can consequently mitigate ROS levels.

Pyruvate kinase muscle isozyme M2 (PKM2) is an isoenzyme that catalyzes the conversion of phosphoenolpyruvate and ADP into pyruvate and ATP. The absence of enzymatic activity from PKM2 diverts glycolytic intermediates into the pentose phosphate pathway (PPP), subsequently resulting in the accumulation of NADPH and the neutralization of reactive oxygen species (ROS) (Anastasiou et al. 2011).

Cell clustering is a mechanism through which detached cancer cells can increase their survival rate. It has been reported that a process of cell clustering following the loss of detachment induces hypoxia-mediated mitophagy (Labuschagne et al. 2019). This process suppresses mitochondrial ROS and removes damaged mitochondria. Conversely, cell clustering is disabled by increased mitochondrial ROS levels, resulting in cell death. Mitophagy induced by extracellular-matrix detachment downregulates NADPH, which in turn increases ROS formation and, consequently, cell death. The divergent responses to mitophagy remain unclear and require further study; however, the only reliable conclusion is the beneficial removal of damaged mitochondria.

#### Circulating tumor cells

It has been reported that neoplastic cells might disseminate from primary tumors during the initial phases of cancer progression (Klein 2013). Circulating tumor cells might serve as precursors to metastases; however, in the bloodstream, they are subjected primarily to harmful shear stress, which can lead to their death through anoikis, a form of programmed cell death triggered by the loss of cell adhesion. Only a small proportion of these cells manage to establish strong interactions with platelets, neutrophils, macrophages, myeloid-derived suppressor cells (MDSCs), or cancer-associated fibroblasts (CAMs), enabling them to evade the immune response, increase their survival, and increase their metastatic potential (Rodrigues And Vanharanta 2019). Such heterotypic clustering has been observed across multiple cancer types, suggesting their potential as compelling therapeutic targets (Yang et al. 2024).

Recently, presented results indicate that the interplay between circulating tumor cells and adverse conditions in the blood microenvironment plays a crucial role in the processes of adhesion to endothelial cells, tissue invasion, and tumor metastasis (Lin et al. 2021). Compared with their original state, tumor cells that enter the bloodstream encounter significantly elevated partial oxygen pressure. During their migration into the circulatory system, these cells may also face ROS-enhanced oxidative stress (Nieto et al. 2016). While melanoma cells metastasizing through the blood are reportedly dependent on ferroptosis, lymph, on the other hand, experiences lower levels of oxidative stress and protects metastatic cells from ferroptosis, thereby increasing their capacity to endure during metastatic events through the blood (Ubellacker et al. 2020).

Increased ROS levels in the prostate and lungs originating from circulating cells induce the expression of β-globin, a subunit of hemoglobin that is abundantly expressed in circulating tumor cells. Depletion of β-globin in circulating tumor cells did not affect the growth of primary tumors; however, it significantly increased apoptosis and reduced lung metastasis following ROS exposure (Zheng et al. 2017). Thus, depletion of β-globin in cancer cells mediates a cytoprotective effect during the metastatic process.

Increased ROS levels in tumor cells are accompanied by the inhibited function of protein tyrosine phosphatases (PTPs), phosphatase and tensin homolog (PTEN), and MAPK phosphatases. This inhibition enhances the MAPK–ERK, PI3K–PKB/Akt, and PKD-NF-kB signaling pathways in a manner that is specific to the cell type (Moloney And Cotter 2018).

To capitalize on the proliferative advantages conferred by elevated ROS levels while reducing the risk of senescence or apoptosis, tumor cells increase the expression of antioxidant transcription factors and/or modify their metabolic processes to increase the production of NADPH and the de novo synthesis of glutathione through various mechanisms. In addition to enhancing antioxidant mechanisms to balance and maintain oxidative stress, tumor cells may activate prosurvival and antiapoptotic signaling pathways.

Breast and lung cancer cells can exploit redox-sensitive transient receptor potential cation channel subfamily A member 1 (TRPA1) to trigger calcium signaling, thereby activating the ERK and PI3K–PKB/Akt pathways (Takahashi et al. 2018). TRPA1 plays a vital role in the survival of inner cells, which results in increased ROS accumulation. Furthermore, TRPA1 contributes to resistance to chemotherapies that generate ROS, while the inhibition of TRPA1 leads to a reduction in tumor growth and an increase in sensitivity to chemotherapy. This activation subsequently results in the upregulation of myeloid cell leukemia 1 (MCL-1), a prosurvival member of the Bcl-2 family of proteins that regulate apoptosis and play a role in balancing oxidative stress. In addition, it has been reported that TRPA1 is upregulated by NRF2 and enhances tolerance to oxidative stress in tumor cells (Takahashi et al. 2018).

## ROS-mediated inhibition of metastasis

It has been reported that subcutaneous administration of metastatic melanomas is markedly more effective than intravenous injection in terms of tumor formation (Piskounova et al. 2015). This was later explained by treatment with the antioxidant N-acetylcysteine, which resulted in the exposure of circulating melanomas to significantly greater levels of oxidative stress than did subcutaneous tumors. Thus, the bloodstream is characterized by a strongly prooxidant environment, hindering metastasis. The survival of metastasizing tumor cells in the bloodstream and their invasion potential are facilitated by upregulated NADPH enzymes (Piskounova et al. 2015). NADPH-mediated support of metastasis is associated with the oxidative pentose phosphate pathway (oxPPP), which plays a key role in redox status and various aspects related to cancer cell viability. These results indicate that oxidative stress is elevated in metastasizing cells relative to that in subcutaneous tumors, which appears to hinder distant metastasis. Nevertheless, it is not known whether oxidative stress also inhibits the initiation and early development stages of primary cutaneous melanomas. Consequently, transient oxidative stress may arise during the formation of primary tumors, in addition to its role in limiting distant metastasis during later stages of cancer progression (Piskounova et al. 2015).

In agreement with these studies, some animal studies have indicated that antioxidants may increase the risk of cancer and accelerate the development of primary lung tumors (Le Gal et al. 2015a, 2015b).

In another related study, briefly outlined above, malignant cells, such as those found in melanoma, frequently spread locally via the lymphatic system before being disseminated systemically through the bloodstream. Melanoma cells residing in lymphatic fluid encounter reduced oxidative stress and exhibit a greater propensity for metastasis than their counterparts in the circulatory system do (Ubellacker et al. 2020). Compared with those disseminated via the lymphatic system, cells that spread through the bloodstream are dependent on the ferroptosis inhibitor GPx4. Both the antioxidant N-acetylcysteine and the water-soluble vitamin E derivative Trolox significantly enhanced the migratory and invasive capabilities of human malignant melanoma cells without affecting their proliferation rates. These antioxidants also increased the ratio of reduced to oxidized glutathione in melanoma cells and lymph node metastases, with increased migration being dependent upon the synthesis of new glutathione. In addition, these two antioxidants activate the small guanosine triphosphatase (GTPase) protein RhoA and inhibit downstream RhoA signaling, effectively suppressing the migration induced by antioxidants. These findings highlight a previously unrecognized role of antioxidants and the glutathione system in the progression of malignant melanoma.

The metastatic potential of cancer cells depends on their metabolic heterogeneity (Tasdogan et al. 2021; Luo et al. 2016). The hypoxic area of primary melanoma tumors presents an increased level of monocarboxylate transporter-1 (MCT1), which is used to transport and release lactate (Tasdogan et al. 2020). Increased levels of MCT1 facilitate resistance to oxidative stress and improve cell survival in the bloodstream by promoting lactate uptake, which in turn lowers the NAD^+^/NADH ratio and pH. These changes upregulate the glucose-oxidizing pentose phosphate pathway (PPP), generating the NADPH necessary to alleviate the effect of oxidative stress (Chen et al. 2019). Taken together, these findings suggest that the upregulation of MCT1 may play an important role in the establishment of an oxidative stress-resistant phenotype characterized by increased survival of metastatic cells in the bloodstream and the ability to metastasize (Godet et al. 2019).

## Dual function of antioxidant activity

Advanced-stage tumors are equipped with mechanisms of adaptation to increased levels of oxidative stress. High ROS levels prompt cancer cells to upregulate NADPH oxidase in various ways and to hyperactivate the Nrf2 pathway, resulting in enhanced antioxidant systems (Chio et al. 2017). The dichotomous role of low-molecular-weight antioxidants and antioxidant enzymes in cancer underscores their regulation, which depends on the specific context throughout various phases of tumor progression.

### Compartmentalization and flux of antioxidants in cancer cells

While the subcellular compartmentalization and transport of ROS and antioxidants in healthy cells are relatively well-characterized, this is not the case in tumors (Hecht et al. 2024). Above all, knowledge about transport in and out of organelles and the localization of their synthesis is considerably limited. A significant amount of cellular reduced glutathione (GSH) is localized within the cytosol; however, it is also present in various organelles, such as mitochondria, peroxisomes, the endoplasmic reticulum, and the nucleus (Lash 2006). Owing to its critical function in maintaining redox homeostasis within cells, glutathione metabolism is often upregulated in numerous cancer types to mitigate oxidative stress and facilitate proliferation and metastasis. It has been reported that GSH is imported into mitochondria to sustain electron transport chain function (Wang et al. 2021). The mitochondrial and nuclear reservoirs of GSH are crucial in safeguarding DNA against damage induced by oxidative stress. In contrast to the reduced state in the majority of cellular organelles, GSH in the endoplasmic reticulum occurs either in its oxidized form or in the form of GSH‒protein complexes. The Sec61 protein-conducting channel has been identified as a central component of the GSH translocation apparatus of the endoplasmic reticulum (ER) membrane (Ponsero et al. 2017). The importance of the endoplasmic reticulum in tumor cells has been associated with an abnormal redox state, which is likely attributable to the protein folding processes occurring within this organelle. This finding is in agreement with the significantly increased levels of oxidized glutathione in the endoplasmic reticulum compared with those in other organelles.

A study of the linkage between GSH and NADPH oxidase in tumor cells revealed that NADPH-mediated support of GSH levels and proper protein folding in the endoplasmic reticulum (Gansemer et al. 2020). GSH-independent enzymes such as peroxiredoxins play important roles in redox homeostasis in the endoplasmic reticulum of tumor cells.

The molecular mechanism of NADPH homeostasis in cancer cells is complex and involves several key pathways and enzymes (Ju et al. 2020). Increased oxidative stress in cancer cells upregulates NADPH oxidase, which results in distinct and independent fluxes between the cytosol and mitochondria (Niu et al. 2023). The primary sources of NADPH in tumor cells characterized by disrupted mechanisms involve (i) the pentose phosphate pathway (PPP), which is essential for maintaining cytosolic NADPH levels; (ii) NAD kinases (NADKs), which facilitate the conversion of NAD(H) to NADP(H) through de novo synthesis, with cytosolic and mitochondrial NADK; and (iii) folate-mediated one-carbon metabolism. In addition, (iv) the isocitrate dehydrogenases IDH1 and IDH2, which are located in the cytosol and mitochondria, respectively, are involved in NADPH production, and (v) glutamine metabolism is involved in NADPH generation through the glutamate dehydrogenases GDH1 and GDH2 in mitochondria.

### Dual role of antioxidant enzymes in cancer

Cancer cells exhibit a notable increase in ROS in specific compartments, which can arise from various factors, such as the activation of oncogenes coupled with the inactivation of tumor suppressor genes. A critical characteristic of these tumor cells is their capacity to cope with elevated levels of ROS, thereby facilitating their survival under detrimental microenvironmental conditions. This is often achieved through the activation of the transcription factor nuclear factor erythroid-2-related factor 2 (NRF2), which subsequently triggers the expression of various antioxidant enzymes, including superoxide dismutases (SODs), peroxiredoxins (Prxs), catalase (CAT), and glutathione peroxidases (GPxs). SODs effectively convert O_2_^•—^ into H_2_O_2_ and O_2_. Catalase and GPx facilitate the reduction of H_2_O_2_ to H_2_O (Bizon et al. 2023). These antioxidant enzymes represent the first line of defense and not only transform two main ROS: O_2_^•—^ into H_2_O_2_ and consequently H_2_O_2_ to H_2_O, but also prevent the formation of damaging ^•^OH via the catalytic decomposition of H_2_O_2_ (the Fenton reaction).

#### Superoxide dismutases (SODs)

Mn-SOD (SOD2), a manganese superoxide dismutase, is localized in mitochondria and has dual functions, acting as a tumor suppressor and promoter. Both of these states are largely attributed to their capacity to scavenge mitochondrial superoxide and regulate the levels of H_2_O_2_. Post-translational modifications and the tumor microenvironment influence the activity and regulation of SOD2 in cancer cells (Kim et al. 2017). The transcription of SOD2 is regulated depending on its role in cancer. Dysregulation of SOD2 expression and activity is associated primarily with the process of cancer development and progression. SOD2 plays a dichotomous role as a tumor suppressor enzyme in the early stages of tumorigenesis while simultaneously acting as a tumor promoter in the process of metastasis. The low occurrence of mutation in cancer cells indicates that SOD2 is highly adaptable. SOD2 expression is modulated by stress response pathways such as NF-κB in response to an altered tumor microenvironment. The release of TNF induces oxidative stress, which triggers SOD2 expression to promote cellular survival and migration within the altered environment (Psaila And Lyden 2009). The observed regulation of SOD2 by cytokines and stress response transcription factors confirms the direct involvement of signals (paracrine or autocrine) originating from the TME. Increased expression of SOD2 has been attributed to tumor-associated fibroblasts of ovarian cells, representing the key component of the tumor microenvironment (Zhang et al. 2018).

Cu, Zn-dismutase (SOD1) is localized in the cytoplasm, the intermembrane space of the mitochondria, and the nucleus. Elevated levels of ROS in cancer cells activate the antioxidant defense system, notably human SOD1 (hSOD1), which is overexpressed in numerous cancers. The overexpression of SOD1 is crucial for tumorigenesis and is mediated by the mammalian target of the rapamycin complex 1 (mTORC1) signaling pathway.

The mTORC1‒SOD1 axis plays a significant role in maintaining the redox equilibrium of cancer cells in response to fluctuating nutritional conditions (König et al. 2024). Undernourished cells during the early stages of carcinogenesis are characterized by inactivated mTORC1 and consequently by overexpressed SOD1. Inhibition of SOD1 can induce the apoptosis of cancer cells, suggesting the potential for novel anticancer treatments.

SOD1 is significantly overexpressed in both non-small cell lung cancer (NSCLC) cell lines and tissues (Liu et al. 2020a, 2020b). This increased expression of SOD1 appears to increase cell proliferation and metastasis, potentially by facilitating cell cycle progression and inhibiting apoptotic processes.

Extracellular SOD (EC-SOD, SOD3) is the only SOD that functions in the extracellular space. EC-SOD plays a special role in cellular signal transduction. Its role in cancer has rarely been studied (Griess et al. 2017). These results suggest that the behavior of SOD3 in cancer is different from that of SOD1 and SOD2. Comprehensive oncology testing, including molecular profiling of cancer patients (Oncomine), revealed significant downregulation of SOD3 expression in a wide range of cancers, including lung, breast, and head and neck cancers, with the highest number of cases meeting the thresholds being breast cancer (Kim et al. 2014). Since SOD3 is a secreted protein, various studies have examined its activity in the serum/plasma of cancer patients compared with healthy individuals. The results of such studies are largely inconsistent. These authors reported a notable correlation between suppressed expression of SOD3 and decreased survival rates in cancer patients, indicating that the dysregulation of the extracellular redox balance may influence a favorable microenvironment for cancer development.

#### Catalase

Catalase is a crucial antioxidant enzyme that facilitates the breakdown of H_2_O_2_ into O_2_ and H_2_O (reaction 5). While catalase is primarily responsible for eliminating elevated levels of H_2_O_2_, glutathione peroxidases and peroxiredoxins degrade low concentrations of H_2_O_2_ (Halliwell and Gutteridge 2015). Like other antioxidant enzymes, catalase is altered in cancer cells, highlighting the significant and dualistic role of this enzyme in tumorigenesis. Catalase has a protective function against the initiation and advancement of tumors by mitigating the accumulation of harmful oxidants. Its downregulation in certain cancers manifests the dichotomous role of catalase in cancer and, conversely, markedly elevated expression in specific malignancies, such as acute myeloid leukemia (AML) (Galasso et al. 2021). Downregulation of catalase has also been observed in lung, pancreatic, prostate, and skin cancers. Increased catalase expression has been observed in gastric cancer, gliomas, and aggressive skin cancer–melanomas.

The molecular mechanism of the regulation of catalase in cancer is not yet fully understood; however, it is affected by genetic and epigenetic alterations, transcriptional and post-transcriptional regulation, and post-translational modifications (Cavallini et al. 2018).

Catalase expression is regulated primarily at the transcriptional level by various factors that can either increase or inhibit the activity of the promoter. Catalase expression was downregulated by the WT1 (Wilms'tumor 1) transcription factor and the early growth response (EGR) transcription factor. Inactivation of the promoter occurs through the disruption of competition with the transactivating function of specificity protein 1 (Sp1) (Nenoi et al. 2001). Positive regulators of catalase expression include, for example, Forkhead transcription factor (FOXO) family members and STAT3 transcription factors.

Epigenetic modifications are associated with altered catalase gene expression. The available data confirmed that chromatin remodeling caused primarily by DNA methylation triggers changes in catalase expression, which is fundamental in cancer progression (Galasso et al. 2021). The exposure of hepatocellular carcinoma cell lines to extensive amounts of ROS results in a reduction in catalase promoter activity, which is facilitated by the hypermethylation of CpG island II within the promoter region (Min et al. 2010).

Post-translational modifications also impact catalase activity via protein folding, affecting the stereochemistry of its tetrameric structure. Most significantly, catalase activity is affected by phosphorylation at the Ser167, Tyr231, and Tyr386 residues. Phosphorylation at Ser167 promotes protein tetramerization, and at two other sites, it increases its enzymatic activity.

Like SOD, catalase is also sensitive to oxidation by ROS (Kim et al. 2001). ROS damage the enzyme’s structure and conformational stability and oxidize proline, lysine, arginine, and histidine residues. All these modifications are responsible for the oxidative inhibition of catalase. The inhibition of membrane-bound catalase by superoxide radicals produced by membrane-associated NADPH oxidase (NOX) represents a novel strategy for antitumor therapy.

Catalase activity is rapidly increased by the phosphorylation of various serine and threonine residues by protein kinases A (PKA) and C (PKC) and serine/threonine casein kinase II (Yano And Yano 2002). In addition, the tumor suppressor protein p53 also regulates catalase activity. High levels of p53 suppress catalase activity, increase oxidative stress, and trigger apoptotic cell death (Kang et al. 2013).

In conclusion, catalase, in the context of cancer, reflects the dual functions of ROS, acting as both a tumor suppressor and a prosurvival protein. Contradicting research findings underscore the complexity of catalase in cancer. Whereas certain studies have indicated a decrease in catalase expression in specific cancers, other reports reported that an increase in catalase levels was correlated with tumor advancement and metastasis (Galasso et al. 2021).

#### Glutathione peroxidase

Glutathione peroxidases (GPxs) belong to a family of enzymes that exhibit peroxidase activity. GPxs convert H_2_O_2_ to H_2_O and peroxides (LOOH) into corresponding alcohols (LOH) in the presence of substrate-reduced glutathione (GSH).

Human glutathione peroxidases (GPxs) consist of eight distinct isozymes, designated GPx1 through GPx8. Among these, five isozymes—specifically GPx1, GPx2, GPx3, GPx4, and GPx6—are selenocysteine (Sec)-containing proteins, referred to as SecGPx. In contrast, the remaining three isozymes, GPx5, GPx7, and GPx8, are characterized as cysteine-containing proteins (CysGPx), where the active site selenocysteine is substituted with cysteine. Each isozyme exhibits variations in molecular structure, compartmentalization, substrate specificity, enzymatic properties, and biological roles (Zhao et al. 2022).

GPx1 is the predominant isoform of glutathione peroxidase. The GPx1 gene is subject to regulation at multiple levels, including transcriptional, post-transcriptional, and translational processes. Notably, GPx1 is intricately linked to tumorigenesis, primarily because of its function in the detoxification of hydroperoxides. GPx1 is often found to be elevated in numerous cancer types, yet it plays complex and opposing roles, acting both as a tumor suppressor and a promoter in various malignancies. Furthermore, GPx1 is involved in multiple signaling pathways that influence tumor biological behaviors, such as cell proliferation, apoptosis, invasion, immune response, and resistance to chemotherapy (Ekoue et al. 2017).

Various studies have investigated the relationship between a functional genetic polymorphism of the GPx1 gene, specifically Pro198Leu, and the risk of cancer across diverse populations. The subjects of these studies were breast (Hu et al. 2010), bladder (Men et al. 2014), prostate (Arsova-Sarafinovska et al. 2009), lung (Raaschou-Nielsen et al. 2007), leukemia (Bănescu et al. 2014), and colon (Hansen et al. 2005) cancer patients. It has been concluded that the GPx1 Pro198Leu polymorphism may increase cancer risk by disrupting the equilibrium of antioxidants.

In the majority of cancers, GPx1 tends to act as a tumor promoter by influencing tumor cell proliferation, invasion, migration, and apoptosis; the immune response; and sensitivity to therapeutic agents (Wei et al. 2020; Kalinina 2024). Compared with those in adjacent normal tissues, significantly elevated levels of GPx1 in tumor tissues have been observed in glioblastoma multiforme, renal papillary cell carcinoma (KIRP), acute myeloid leukemia (AML), low-grade glioma (LGG), ovarian serous cystadenocarcinoma, pancreatic adenocarcinoma, skin melanoma, testicular germ cell tumor, thyroid cancer, and endometrial cancer (Wei et al. 2020). GPx1 may exhibit contrasting functions across various cancer types. A progressive decrease in GPx1 expression has been observed in pancreatic cells transitioning from a healthy pancreas to chronic pancreatitis and ultimately to pancreatic cancer (Cullen et al. 2003).

In esophageal cancer and salivary adenoid cystic carcinoma, GPx1 expression facilitates invasion, migration, proliferation, and resistance to cisplatin. Notably, since NF-κB is known to activate GPx1 transcriptionally, vitamin D has the potential to inhibit the NF-κB pathway, thereby reducing both GPx1 expression and tumor aggressiveness (Gan et al. 2014; Huang et al. 2016).

GPx2, along with GPx1, plays an important role in mitigating oxidative damage through the reduction of hydroperoxides. Notably, GPx2 is frequently upregulated in various types of tumor cells and is associated with a poor prognosis in cancer patients (Hashinokuchi et al. 2024). Increased expression of GPx2 in male lung adenocarcinoma patients has been associated with reduced overall survival rates. GPx2 overexpression has been shown to activate the Wnt/β-catenin and epithelial‒mesenchymal transition (EMT) pathways, which are associated with prostate cancer (Yang et al. 2022). GPx2 overexpression has been implicated in the metastasis of lung and colorectal cancers. Conversely, a decrease in GPx2 has been detected in bladder, esophageal, and breast tumors and has also been associated with poorer prognoses (Ren et al. 2022). GPx2 knockout in murine models has been associated with intestinal tumorigenesis and increased susceptibility to skin cancer following irradiation (Brigelius-Flohé And Kipp 2009).

GPx3 also has dual functions in cancer. GPx3 acts as a prosurvival factor in myeloid leukemia. In contrast, it functions as a tumor suppressor in gastrointestinal and pulmonary cancers (Liu et al. 2019; He et al. 2023). The protective role of upregulated GPx3, manifested by the suppression of the growth of melanoma cells via the ROS mechanism, has been attributed to the inhibition of HIF1α, an important factor frequently elevated in various malignancies (Yi et al. 2019). Tumors such as breast, head and neck, renal, gastrointestinal, and lung cancers exhibit markedly diminished expression of GPx3, which is attributed to epigenetic mechanisms, including methylation and histone modifications (Hu et al. 2023).

Owing to its unique amino acid sequence and stereochemistry, GPx4 has the capacity to reduce complex lipid hydroperoxides even when they are integrated into biological membranes (Ursini et al. 1982).

The synthesis and expression of GPx4 can be modulated through various mechanisms, including transcriptional regulation, translational control, post-translational modifications, and epigenetic alterations. Like other GPx family members, GPx4 has dual roles in tumorigenesis. GPx4 in tumor tissues has been reported to be markedly elevated compared with that in normal tissues, as evidenced in various cancers, including gastrointestinal, renal, lung, prostate, and thyroid cancers (Wei et al. 2022). This observation suggests that GPx4 could function as an oncogene. GPx4 plays a close role in cancer and functions as the primary inhibitor of a relatively recently discovered new type of intracellular iron-dependent form of cell death termed ferroptosis (Zhang et al. 2024). GPx4 has an important function in the cytosol as a key inhibitor of ferroptosis and various mechanisms that control vulnerability to ferroptosis. Therefore, the inhibition of GPx4 can result in cellular apoptosis, which may trigger inflammatory responses in the tumor microenvironment, potentially affecting tumor development and progression (Zhou et al. 2024). Focusing on GPx4 as a therapeutic target in anticancer therapy represents a potentially effective approach for promoting ferroptosis and eliminating cancer cells that are resistant to conventional therapies.

The roles of GPx5, GPx6, GPx7, and GPx8 in the context of cancer are relatively rare, in contrast with the extensive studies conducted on GPx1—GPx4. Notably, GPx5 is an enzyme predominantly expressed in the epididymis and contains cysteine residues in place of selenocysteine at its active site (Taylor et al. 2013). GPx5 is crucial for preserving the microenvironment within the epididymis, safeguarding sperm against oxidative stress, and ensuring the structural integrity of DNA. In humans, GPx6 functions as a selenoprotein characterized by the presence of selenocysteine at its active site. It has been reported that GPx5 and GPx6 are downregulated in highly aggressive and invasive triple-negative breast cancer cells compared with healthy breast cells (Rusolo et al. 2017).

GPx7 is located within the lumen of the endoplasmic reticulum (ER), whereas human GPx8, which is anchored to the ER, functions as a type II transmembrane protein. In this configuration, the cysteine residue that is essential for its catalytic activity resides in the ER lumen (Nguyen et al. 2011). GPx7 is an important regulator of redox homeostasis under conditions of oxidative stress. The disturbed regulation of GPx7 has been implicated in various diseases, notably cancer. Research indicates that varying levels of GPx7 expression are significantly associated with the initiation and progression of several tumors. GPx7 overexpression has been observed in hepatocellular carcinoma (Guerriero et al. 2015) and contributes to glioma progression via several pathways, such as the cell cycle pathway (Yao et al. 2021). It has been proposed that Gpx7 expression may be a valuable biomarker of lower grade gliomas.

Dysregulated expression of GPx8 has been reported in various tumors, which may emphasize its potential as a therapeutic target in cancer treatment. GPx8 has been shown to sustain the invasiveness of breast cancer cells via the IL-6/STAT3 signaling pathway (Khatib et al. 2020) and acts as a prognostic biomarker of gliomas and gastric cancer (Zhang et al. 2022). Elevated expression of GPx8 is correlated with decreased patient survival and the establishment of an immunosuppressive microenvironment (Bai et al. 2024). The suppressed activity of GPx8 has been shown to inhibit the development of renal cell tumors by modulating nicotinamide N-methyltransferase (NNMT) (Nguyen et al. 2023).

### Dual role of low-molecular-weight antioxidants in cancer

Many low-molecular-weight antioxidants, such as vitamin C, vitamin E, carotenoids, and flavonoids, exhibit efficient ROS-scavenging properties under in vitro conditions (Sznarkowska et al. 2017). However, clinical trials evaluating the antioxidant/health-beneficial properties of antioxidants are less promising. Some antioxidants may, under certain conditions, behave as prooxidants.

#### Vitamin C (ascorbic acid)

Vitamin C effectively scavenges ROS and RNS, potentially mitigating oxidative damage to crucial biological macromolecules, including DNA, lipids, and proteins. In addition, ascorbate has the capacity to reduce free redox-active transition metal ions, such as copper and iron. The prooxidant properties of ascorbic acid include its ability to reduce ferric ions to ferrous ions (Fe^3+^ → Fe^2+^), subsequently producing dehydroascorbic acid. Ascorbic acid enhances the redox cycle between Fe^3+^ and Fe^2+^ (Fe^3+^ ↔ Fe^2+^) as a catalyst in the Fenton reaction, increasing the degree of damage to hydroxyl radicals (Timoshnikov et al. 2020).

The antioxidant and redox-metal-reducing properties of vitamin C are associated predominantly with in vitro conditions. However, the behavior under in vivo conditions is more complex, and vitamin C can undergo a switch from antioxidant to prooxidant properties depending on the conditions (concentration, presence of redox metals, and other factors). Healthy volunteers (16 females and 15 males) were supplemented with 500 mg of vitamin C per day for 6 weeks, and consequently, the levels of two representative biomarkers of oxidative damage, 8-oxoguanine and 8-oxoadenine, in lymphocyte DNA were evaluated via chromatography‒mass spectrometry (GC‒MS) (Podmore et al. 1998). Interestingly, while supplementation with vitamin C resulted in a marked decrease in 8-oxoguanine, the level of 8-oxoadenine increased compared with the baseline and placebo values (Dizdaroglu 1994). Based on the results of this study, supplementation with vitamin C up to 500 mg/day appears to be safe and predominantly antioxidant in nature. In addition, not only the dose of the antioxidant but also the target tissue, the partial pressure of oxygen, and other factors may affect the switch from antioxidant to prooxidant behavior.

Several clinical studies have established a correlation between increased dietary intake of vitamin C and a reduced risk of stomach cancer (Liu And Russell 2008). A detailed analysis revealed that vitamin C can inhibit the production of carcinogenic N-nitroso compounds in the stomach (Tsugane And Sasazuki 2007).

Decreased levels of vitamin C characterize infection with *Helicobacter pylori* (*H. pylori*) and have been established as factors increasing the risk of stomach cancer incidence. Some trials reported that cotherapy consisting of vitamin C in standard *H. pylori* treatment may be beneficial in mitigating the risk of gastric cancer (Chuang et al. 2007). The beneficial effect of vitamin C has been attributed to the inhibitory effect of urease, facilitating the survival of *H. pylori* (Krajewska And Brindell 2011).

The effect of vitamin C intake on colon cancer incidence has been studied in more than 600 000 individuals (Park et al. 2010). The results revealed that dietary intake of vitamin C did not affect the incidence of colon cancer, whereas dietary intake plus supplementation with vitamin C reduced the risk of colon cancer by nearly 20%.

**Fig. 9:**
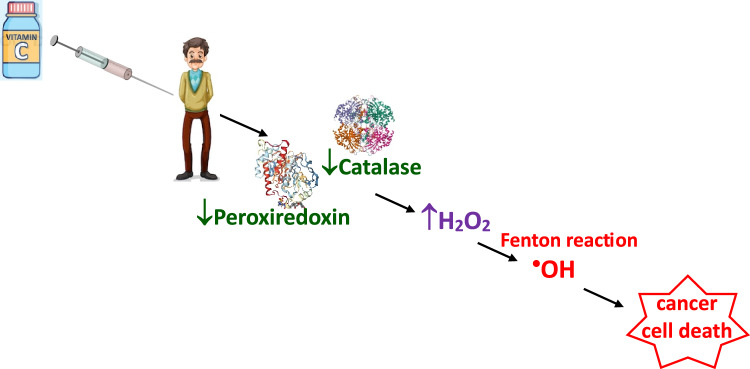
Intravenous administration of vitamin C oxidizes the hydrogen peroxide-converting enzyme peroxiredoxin. In cancer cells, the concentration of the hydrogen peroxide-converting enzyme catalase is reduced. Suppressed levels of both peroxiredoxin and catalase result in an increased level of hydrogen peroxide, which can serve as a precursor for hydroxyl radicals through the Fenton reaction, potentially causing damage to cancer cells

In the past several decades, interest in the prooxidant role of ascorbate as a cochemotherapy agent has increased (Nauman et al. 2018a, 2018b; Gorkom et al. 2019). The therapeutic benefits of intravenous ascorbate are largely attributed to its prooxidant characteristics, which can induce damage to malignant cells. A key mechanism of the prooxidant action of ascorbate involves the oxidation of the hydrogen peroxide scavenging enzyme peroxiredoxin-2. Oxidized peroxiredoxin 2, together with lowered levels of catalase in cancer cells, leads to increased levels of hydrogen peroxide, a source of hydroxyl radicals generated via the Fenton reaction, potentially causing damage to cancer cells (Fig. 9) (Jomova et al. 2024).

#### Vitamin E (α-tocopherol)

Vitamin E is a fat-soluble antioxidant that terminates peroxyl radicals responsible for the lipid peroxidation process. As outlined above, the radical form of vitamin E (α-tocopheroxyl radical, α-toc^•^) can be regenerated by vitamin C.

Oxidative-induced DNA damage by ROS can cause mutations that may play a role in the development of cancer (Dizdaroglu 2012). As an efficient ROS scavenger, vitamin E has been proposed to play a role in preventing cancer by protecting cells from oxidative damage. However, numerous clinical studies have failed to confirm the protective effect of vitamin E on the incidence of lung or breast cancer. The VITamins And Lifestyle (VITAL) trial, which employs over 70,000 individuals, evaluated the effects of long-term supplemental vitamin use (a decade) on the risk of lung cancer (Slatore et al. 2008). No significant associations between the intake of multivitamins, vitamins C and E, or folate and lung cancer risk were found. Notably, the use of supplemental vitamin E (100 mg/day) among current smokers, but not former smokers, was correlated with an 11% increase in the risk of lung cancer.

As part of the alpha-tocopherol, beta-carotene cancer (ATBC) prevention trial, the impact of synthetic α-tocopherol supplementation (50 mg/day) revealed a 32% decrease in the incidence of prostate cancer (Heinonen et al. 1998). However, during the 18-year follow-up period postintervention, no significant differences in prostate cancer incidence were observed between those who received α-tocopherol and those who did not (Virtamo et al. 2014; Higdon et al. 2024).

The combined effect of selenium and vitamin E on prostate cancer risk was investigated (Kristal et al. 2014). Surprisingly, selenium supplementation was found to be ineffective for individuals with low selenium levels, whereas it was associated with an elevated risk of high-grade prostate cancer in those with high selenium levels. In addition, vitamin E supplementation increases the risk of prostate cancer in men with a low selenium status. Therefore, men should refrain from selenium or vitamin E supplementation at doses that surpass the recommended dietary allowances.

In conclusion, the majority of randomized controlled trials have not demonstrated a significant advantage of vitamin E supplementation in preventing cancer.

#### Carotenoids

Carotenoids comprise more than 800 naturally occurring colored pigments synthesized by plants, bacteria, and algae. Fruits and vegetables contain approximately 50 carotenoids that are beneficial for human health (Higdon et al. 2023). Carotenoids play crucial roles as antioxidants by scavenging singlet oxygen generated during photosynthesis. Lycopene is among the most potent singlet oxygen quenchers within the carotenoid family; however, its importance in humans remains unambiguous. In vitro studies have indicated that carotenoids can prevent the lipid peroxidation process under specific conditions; however, their effects on human physiology seem to be more intricate (Jomova And Valko 2013).

The most prominent trial of the unexpected behavior of carotenoids was a large-scale randomized placebo-controlled trial examining the impact of supplemental β-carotene, known as the ATBC Trial (Kataja-Tuomola et al. 2010). This α-tocopherol/β-carotene cancer prevention study spanned 6 years and involved 29,000 male smokers who received either β-carotene, α-tocopherol, or a combination of both. The findings revealed a 16% increase in the incidence of lung cancer among participants taking β-carotene supplements. A study aimed at clarifying this paradoxical result revealed that high-dose β-carotene supplementation significantly elevated the levels of β-carotene oxidation products (Veeramachaneni et al. 2009). This study revealed that the switch between the antioxidant and prooxidant properties of carotenoids depends on the conditions and site of action (Terao 2023). In this case, the critical conditions are the concentration of carotenoids and the partial pressure of oxygen (high in the lungs), where peroxyl radicals derived from carotenoids can be formed.

Prostate cancer ranks as one of the most common cancers affecting men. It has been proposed that a diet high in lycopene is linked to a notable decrease in the risk of prostate cancer (Giovannucci 2002). A meta-analysis consisting of 10 case cohorts and 2 prospective cohort studies revealed no notable correlation between dietary intake of lycopene and prostate cancer risk (Wang et al. 2015).

Two minor randomized controlled trials over 6 months evaluated the impact of lycopene supplementation (30–35 mg/day) on precancerous prostate lesions. This study was performed alone or in combination with selenium (55 mg/day) and green tea catechins (600 mg/day). The findings of this study indicated no significant advantage in delaying the progression of prostate cancer (Gann et al. 2015; Gontero et al. 2015).

A meta-analysis revealed no significant relationship between carotenoid intake and breast cancer risk; the only exception, a 5% decrease in the incidence of breast cancer, was reported for an additional intake of 5 mg/day of β-carotene (Aune et al. 2012).

**Fig. 10:**
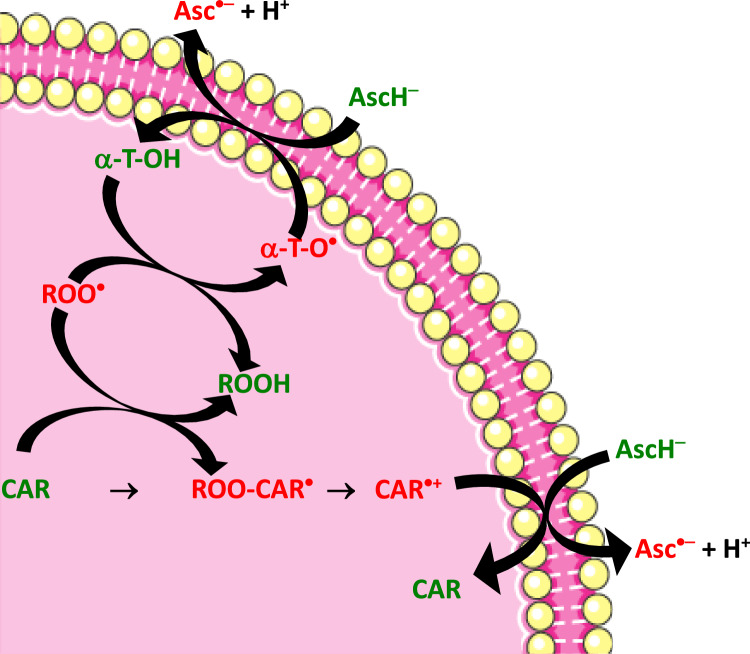
Interplay between vitamin C (AscH^—^), ascorbyl radical (Asc^•—^), vitamin E (α-T-OH), tocopheroxyl radical (α-T-O^•^), carotenoids (car) and carotenoid radical (car^•+^). Adapted from Jomova et al. 2024

Another study demonstrated an inverse relationship between blood levels of β-carotene/α-carotene and the risk of estrogen receptor-negative (ER-) breast tumors. However, no such correlation was observed for estrogen receptor-positive (ER +) tumors (Eliassen et al. 2012). The interplay between vitamins C and E and carotenoids is outlined in Fig. 10**.**

#### Flavonoids

Flavonoids represent a group of more than 5000 compounds that play crucial roles in plant biology. Flavonoids frequently occur in edible plants and foods that are bound to sugar molecules (β-glycosides) (Higdon et al. 2016). The basic skeleton of flavonoids consists of fused A and C rings, with the phenyl B ring attached through its 1′ position to the 2nd position of the C ring (Fig. 2).

Flavonoids are effective ROS scavengers that act predominantly under in vitro conditions. They undergo various transformations in living organisms, resulting in their concentration in blood plasma being very low, typically up to 700 times lower than those of other low-molecular-weight antioxidants, such as ascorbate or carotenoids. Circulating metabolites usually exhibit lower antioxidant activity than their parent molecules do; however, flavonoid metabolites may act as signaling molecules. The key antioxidant activity of flavonoids is manifested by the number of hydroxyl groups and their capacity to chelate redox-active metal ions (copper, iron), which, as free (unbound), can effectively catalyze the formation of damaging radicals (e.g., hydroxyl radicals) via the Fenton reaction. The catalytic activity of chelated redox-metal ions is significantly suppressed.

In addition to their antioxidant properties, flavonoids may, under certain conditions, have prooxidant properties. Metal–flavonoid complexes, such as Cu (II)–kaempferol, exhibit weak DNA intercalating properties, which are essential for conferring the anticancer activity of these substances (Simunkova et al. 2021).

In addition, it has been reported that the prospective anticancer effect of flavonoids can be attributed to the increased production of ROS, which may, in the early stages of cancer development, cause damage to proliferating cells. The mild prooxidant properties of Cu (II)–flavonoid complexes might stimulate the activation of antioxidant mechanisms, including the action of antioxidant enzymes and low-molecular-weight antioxidants such as glutathione, thereby functioning as preventive anticancer agents (Jomova et al. 2019).

Various animal models have confirmed that flavonoids can act as inhibitors of chemically induced cancers, including gastrointestinal (Yamane et al. 1996), prostate (Haddad et al. 2006), lung (Yang et al. 1998), and other cancers. In contrast to animal models, observational studies have not confirmed that increased intake of natural flavonoids reduces the risk of cancer in humans (Romagnolo And Selmin 2012).

A robust meta-analysis consisting of more than 30 studies revealed that the intake of total flavonoids and flavonoid subclasses was inversely correlated with the risk of smoking-related cancers of the respiratory tract and proximal portion of the digestive tract (Woo And Kim 2013). No significant correlation between the risk of colorectal cancer and the consumption of flavonoid-rich foods such as oranges, blueberries, and tea (Nimptsch et al. 2016) was found. Positive results from a clinical trial involving Japanese men revealed a notable reduction in prostate cancer incidence following the oral administration of 60 mg/day isoflavones (Miyanaga et al. 2012). Interestingly, this study confirmed that prostate cancer risk was decreased independently of hormone-sensitive mechanisms. More studies must be conducted to determine whether flavonoids positively affect cancer prevention.

## Conclusions

Redox reactions, encompassing electron-transfer reactions, are fundamental to the sustenance of life. To maintain proper functioning of cellular processes, cells rely on coordinated control of the redox system, which includes various input signals that trigger the generation of a redox signal, followed by its transmission to designated target locations. Systems that generate and eliminate reactive oxygen species (ROS) play crucial roles in sustaining the intracellular redox balance, which is essential for mediating redox signaling and governing cellular activities. Physiological concentrations of oxidants (ROS) modulate the functions of kinases, phosphatases, and transcription factors, thereby promoting processes, such as proliferation, differentiation, and migration.

The intricate role of ROS levels in all stages of tumorigenesis fundamentally relies on fine-tuning of their generation and elimination. The onset and progression of cancer exploit gradual elevations in ROS levels. Consequently, tumor cells can employ a range of adaptive mechanisms, effectively maintaining ROS levels within a dynamic range that promotes proliferation while preventing cell death. The initiation period is characterized by gradually increasing oncogene-induced oxidative stress, which triggers enhanced antioxidant defense, frequently involving the upregulation of antioxidant genes regulated by nuclear factor erythroid 2-related factor 2 (NRF2). In addition, this period involves increased NADPH synthesis by redirecting glucose metabolism through the pentose phosphate pathway (PPP).

The current state of knowledge cannot answer questions such as the threshold level of oxidative stress necessary to activate tumor development, the types of ROS that promote tumorigenic/antitumorigenic signaling, critical subcellular localizations where oxidative stress peaks, and other factors. Crucial in terms of oncogenic transformation is the efficiency of oxidative stress-counteracting mechanisms, involving the extent of antioxidant gene expression, increased GSH synthesis, NADPH production, and other defense mechanisms to reverse the critical level of oxidative stress.

While angiogenesis is important in the formation of new vessels and essential for embryonic development, its dysregulation can contribute to various pathologies, including cancer. Recent findings indicate that ROS produced from different and distant sources may “interact” with each other to regulate certain physiological processes, including angiogenesis. The crosstalk between mitochondria and NOX has been termed “ROS-induced ROS release”, which positively affects ROS production at specific cell compartments and consequently triggers beneficial redox signaling to modulate cellular processes. A deeper understanding of such mechanisms may open new avenues for therapeutic interventions in angiogenesis-related cancers.

ROS affect both tumor and mesenchymal stromal cells via the collective response of all cells within the tumor microenvironment (TME). ROS generated by cancer cells facilitate the development of cancer-associated fibroblasts and myofibroblasts, enhancing tumor growth and invasiveness. In addition, ROS can activate tumor-associated macrophages to trigger angiogenic and immunosuppressive functions, which further activate regulatory T cells and suppress the activity of natural killer (NK) cells and effector cytotoxic T cells.

The dichotomy of ROS manifests in premalignant and malignant cells during all stages of tumor development. On one hand, ROS initiate and facilitate the proliferation of already initiated cells during the promotion and progression phases of tumor development. On the other hand, ROS have the potential to induce senescence or apoptosis in neoplastic cells throughout all phases of tumorigenesis, which includes anchorage-independent growth and metastasis. In the context of the activation of various antioxidant systems, cancer cells respond to increased oxidative stress through elevated levels of reduced glutathione and the thioredoxin system, which usually correlate with a poor prognosis and survival.

The (i) initiation stage of tumorigenesis is characterized by increased production of peroxinitrite (ONOO^—^), superoxide (O_2_^•—^) and hydroxyl (^•^OH) radicals and hydrogen peroxide (H_2_O_2_) to accomplish the genetic alterations necessary for independent cellular proliferation. During the (ii) promotion stage, H_2_O_2_, which acts as a signaling molecule, plays a key role in maintaining the proliferation of preneoplastic cells that have developed the ability for independent growth. The contradictory effects of different types of ROS characterize the (iii) progression stage. Protumorigenic activity is mediated by H_2_O_2_, which promotes epithelial-to-mesenchymal transition (EMT) and tumor-associated macrophages, which participate in the formation of the tumor microenvironment. Conversely, O_2_^•—^ which is used by the immune system together with ONOO^−^ dependent mechanisms at all levels of the antitumor immune response, may participate in the killing of tumor cells. The decisive factor will be which of the mechanisms will prevail. This stage is characterized by the local infiltration of tumor cells into surrounding healthy tissues, characterized by growth that does not require anchorage, along with their interaction with mesenchymal stromal and immune cells. (iv) Metastasis is a critical hallmark of cancer and is affected by oxygen homeostasis in the tumor microenvironment and the level of antioxidant systems, depending on the type of tumor. This stage is characterized by the migration of cancer cells through the circulation to establish a distant secondary tumor focus.

As is the case with radicals, a dichotomy is a characteristic feature throughout all stages of cancer for antioxidants as well. A critical characteristic of tumor cells is their capacity to cope with elevated levels of ROS, thereby facilitating their survival under detrimental microenvironmental conditions. This is often achieved through the activation of the transcription factor nuclear factor erythroid-2-related factor 2 (NRF2), which subsequently triggers the expression of various antioxidant enzymes. These antioxidant enzymes represent the first line of defense and not only transform two main ROS, O_2_^•—^ into H_2_O_2_ and H_2_O_2_—into H_2_O but also prevent the formation of damaging ROS.

The relatively rare Mn-SOD (SOD2) gene loss or gene mutation in cancer suggests the flexible regulation of this antioxidant enzyme. The dichotomous role of SOD2 in cancer involves its role as a tumor suppressor enzyme in the early stages of tumorigenesis while simultaneously acting as a tumor promoter in the process of metastasis. SOD2 expression is modulated by stress response pathways such as NF-κB in response to an altered tumor microenvironment. The release of TNF induces oxidative stress, which triggers SOD2 expression to promote cellular survival and migration within the altered environment. The dichotomous role of catalase in cancer is not fully understood and has been attributed to genetic and epigenetic alterations, transcriptional and post-transcriptional regulation, and post-translational modifications.

Even though low-molecular-weight antioxidants, including vitamins C and E, carotenoids, and flavonoids, exhibit efficient antioxidant properties under in vitro conditions, the results of clinical trials are much less convincing. One of the most prominent examples of the dual behavior of low-molecular-weight antioxidants is beta-carotene. The large, α-tocopherol/β-carotene cancer prevention study spanned 6 years and revealed a 16% increase in the incidence of lung cancer among participants taking β-carotene supplements. Later studies reported that high-dose β-carotene supplementation significantly elevated the levels of β-carotene oxidation products, probably β-carotene-derived peroxyl radicals (β-car-OO^•^). This study revealed that the switch between the antioxidant and prooxidant properties of carotenoids depends on the conditions and site of action. In this case, the critical conditions are the concentration of carotenoids and the partial pressure of oxygen (high in the lungs), where peroxyl radicals derived from carotenoids can be formed and cause oxidative damage.

Research is gradually progressing to recognize the critical role of redox homeostasis in cancer. Despite several advances, unresolved issues include the impact of protumorigenic and antitumorigenic mechanisms in different cell types and the mechanisms by which the antioxidant transcription factor network functions at various stages of tumorigenesis. Specifically, it is essential to investigate (i) the critical ROS thresholds that trigger the activation of individual network components and (ii) how these thresholds for specific transcription factors may vary throughout tumorigenesis. Importantly, (iii) the role of stresses such as hypoxia, inflammation, and metabolic stress in tumorigenesis should be explored. Furthermore, it is crucial to explore (iv) the quantitative assessment of oxidative stress levels that promote epithelial‒mesenchymal transition (EMT), especially in the context of the metastatic potential of various tumor cell types. Understanding the redox thresholds that affect EMT and mesenchymal‒epithelial transition (MET) is likely to reveal significant biochemical pathways that determine the metastatic potential of various tumor cell types. Focusing on these aspects, together with the evaluation of the effects of the antioxidant network, may result in the development of more effective therapeutic interventions.
